# Dietary fatty acids promote lipid droplet diversity through seipin enrichment in an ER subdomain

**DOI:** 10.1038/s41467-019-10835-4

**Published:** 2019-07-01

**Authors:** Zhe Cao, Yan Hao, Chun Wing Fung, Yiu Yiu Lee, Pengfei Wang, Xuesong Li, Kang Xie, Wen Jiun Lam, Yifei Qiu, Ben Zhong Tang, Guanghou Shui, Pingsheng Liu, Jianan Qu, Byung-Ho Kang, Ho Yi Mak

**Affiliations:** 1 Division of Life Science, The Hong Kong University of Science and Technology, Hong Kong SAR, China; 2 School of Life Science, The Chinese University of Hong Kong, Hong Kong SAR, China; 3 Biophotonics Research Laboratory, Department of Electronic and Computer Engineering, The Hong Kong University of Science and Technology, Hong Kong SAR, China; 4 Institute of Biophysics, Chinese Academy of Sciences, Beijing, China; 5 Department of Chemistry, The Hong Kong University of Science and Technology, Hong Kong SAR, China; 6 Institute of Genetics and Developmental Biology, Chinese Academy of Sciences, Beijing, China

**Keywords:** Fatty acids, Organelles, Metabolic pathways, Caenorhabditis elegans

## Abstract

Exogenous metabolites from microbial and dietary origins have profound effects on host metabolism. Here, we report that a sub-population of lipid droplets (LDs), which are conserved organelles for fat storage, is defined by metabolite-modulated targeting of the *C. elegans* seipin ortholog, SEIP-1. Loss of SEIP-1 function reduces the size of a subset of LDs while over-expression of SEIP-1 has the opposite effect. Ultrastructural analysis reveals SEIP-1 enrichment in an endoplasmic reticulum (ER) subdomain, which co-purifies with LDs. Analyses of *C. elegans* and bacterial genetic mutants indicate a requirement of polyunsaturated fatty acids (PUFAs) and microbial cyclopropane fatty acids (CFAs) for SEIP-1 enrichment, as confirmed by dietary supplementation experiments. In mammalian cells, heterologously expressed SEIP-1 engages nascent lipid droplets and promotes their subsequent expansion in a conserved manner. Our results suggest that microbial and polyunsaturated fatty acids serve unexpected roles in regulating cellular fat storage by promoting LD diversity.

## Abstract (web summary)

Lipid droplets (LDs) are fat storage organelles that are initiated and expanded by seipins at ER contact sites. Here the authors show that the *C. elegans* seipin ortholog SEIP-1 is recruited to these sites by certain dietary fatty acids to support the expansion of a subset of LDs.

## Introduction

Lipid droplets (LDs) are the primary organelles for fat storage in all eukaryotic cells^1–3^. They accommodate excess energy in the forms of triglycerides (TAG) and cholesterol esters (CE) within a phospholipid monolayer that is enriched in phosphatidylcholine or phosphatidylethanolamine^4,5^. Extensive proteomic analyses revealed a core set of proteins that reside on the surface of LDs, some of which regulate fat deposition and mobilization^6^. Although LDs play a central role for intracellular fat storage, they are known to accommodate additional cargos in a developmental stage- and tissue-specific manner^7–9^. The diversification of LDs is further hinted by the preferential association of metabolic enzymes to subsets of LDs^10,11^. However, the molecular mechanisms that underlie LD diversity are not fully understood.

In the energy-replete state, LDs are closely associated with the endoplasmic reticulum (ER)^12,13^, which can be facilitated by protein–protein interactions or through stable or transient membrane connections^11,14–17^. The physical coupling of the two organelles is required for LD expansion, in part because TAG precursors are made in the ER before they are converted into TAG by DGAT2 at the ER-LD interface^11,14,18,19^. Additional evidence for a fundamental role of the ER in cellular fat storage comes from the discovery that Atlastins regulate LD size^20^. Atlastins are conserved proteins essential for the generation and maintenance of the tubular ER network. Depletion of Atlastins reduces LD size^20^, possibly due to a loss of tubular ER subdomains that are dedicated for ER-LD coupling. However, how such subdomains are maintained and segregated from the rest of the network is unknown.

The seipin protein family plays a conserved role in lipid homeostasis and maintenance of lipid droplet morphology. Mutations in seipin/BSCL2 cause a severe form of congenital generalized lipodystrophy in humans^21,22^. Pleiotropic metabolic defects such as insulin resistance, hypertriglyceridemia, and hepatic steatosis are thought to stem from an almost complete lack of adipose tissues. Accordingly, seipin has been shown to act cell-autonomously to promote adipocyte differentiation^23–25^. Biochemical and structural studies suggest that seipin assembles into homo-oligomers^26–28^. Intriguingly, seipin orthologs were found at ER-LD junctions where it may be responsible for the proper partitioning of lipids and proteins between the ER and LDs^22,29–32^. In addition, loss of seipin function in yeast and *Drosophila* alters the profile of acyl chains in phospholipids^30,33^, which has been proposed to cause gross disturbance to LD morphology, resulting in ‘supersized’ LDs or clusters of abnormally small and misshapen LDs^30,32^. More recently, a role for seipin in the maturation of nascent LDs has been reported^34^. Therefore, the precise localization of seipin appears to be critical for its function.

The phospholipid composition is a major determinant of the structure and function of eukaryotic membrane-bound organelles^35^. The synthesis of phospholipids is in turn dependent on the availability of exogenous and endogenous precursors of head groups and fatty acyl chains in the form of fatty acids^36^. In humans, dietary polyunsaturated fatty acids are readily incorporated into membrane phospholipids. They modulate membrane fluidity and serve as precursors for key immunomodulatory molecules. In addition to the diet, the gut microbiome is another potential source of exogenous fatty acids. For example, bacterial cyclopropane fatty acids are found to be incorporated into phospholipids and triglycerides in human adipose tissue^37^. However, the significance of bacterial fatty acids in eukaryotic membranes is so far obscure.

In this paper, we report that human seipin and its *C. elegans* ortholog SEIP-1 are targeted to a tubular ER subdomain. SEIP-1 is enriched in ER tubules that tightly associate with a subset of LDs. Proper targeting of SEIP-1 is critically dependent on specific polyunsaturated fatty acids (PUFAs) and bacterial cyclopropane fatty acids. Therefore, LDs heterogeneity may originate from fatty acid-driven targeting of distinct protein ensembles.

## Results

### SEIP-1 regulates LD size

**Fig. 1:**
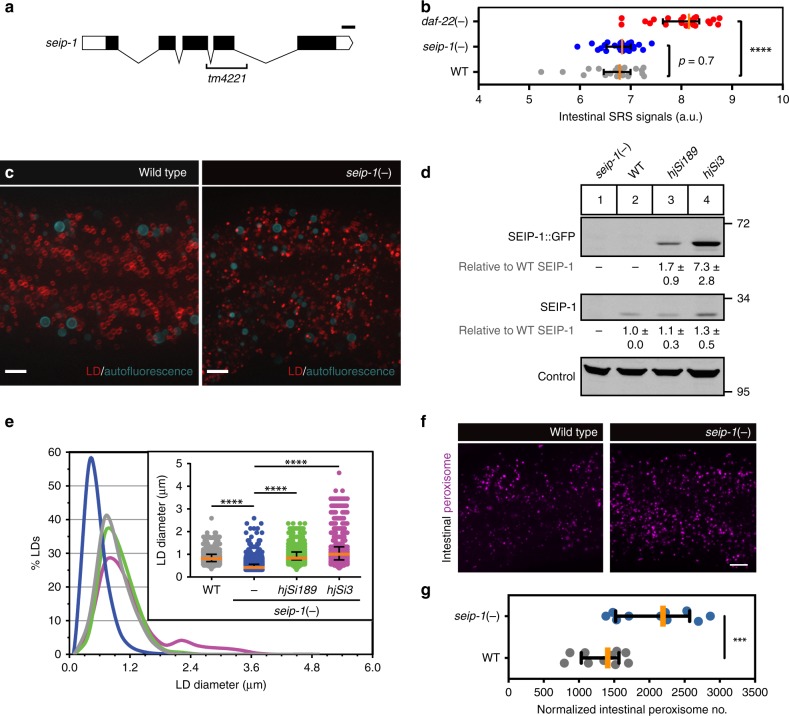
SEIP-1 regulates LD size. **a** Schematic representation of *seip-1*. Exons and untranslated regions are indicated by black and white boxes, respectively. The region deleted in the *tm4221* allele is indicated. Scale bar = 100 bp. **b** Label-free quantification of neutral lipid content of individual animals by Stimulated Raman Scattering (SRS). Number of animals analyzed: wild type (WT) = 22, *daf-22(−)* = 22, *seip-1(−)* = 24. **c** Visualization of LDs using mRuby::DGAT-2 (*hjSi112*) in a WT or *seip-1* (*tm4221*) mutant larval L4 stage animal. mRuby is in red and autofluorescence from lysosome-related organelles (LROs) is pseudocolored cyan. A projection of 7.5 μm z stack centering at the second intestinal segment is shown. **d** The expression levels of SEIP-1::GFP and endogenous SEIP-1 were determined by SDS-PAGE and immunoblotting with anti-SEIP-1 antibodies. A non-specific band at ~95 kDa served as a loading control. The expression level of endogenous SEIP-1 in WT animals served as a reference for normalization of signals in other samples. Mean ± SD is presented from three independent experiments. **e** Frequency distribution of LD diameter. The curve was fitted twice using Fit Spline/LOWESS (20 points in smoothing window, 4000 segments) method based on a histogram with a bin size of 0.3 μm (see Supplementary Fig. 1d, applicable to all subsequent frequency distribution curves). Inset: a scatter plot summarizing LD diameter. Total number of LDs measured: WT = 1680, *seip-1* (*tm4221*) = 3934, *hjSi189*;*seip-1* (*tm4221*) = 2224, and *hjSi3;seip-1* (*tm4221*) = 1209. **f** Visualization of intestinal peroxisomes in a WT or *seip-1* (*tm4221*) larval L4 stage animal. Signals from tagRFP::DAF-22 peroxisomal thiolase were pseudocolored magenta. A projection of 6.5 μm z stack centering at the second intestinal segment is shown. **g** The total number of intestinal peroxisomes in a normalized volume of 80 × 80 × 6.5 μm (*xyz*). *n* = 10 animals. For all fluorescence images, scale bar = 5 μm. For all scatter plots, median with interquartile range is displayed. For all statistics, *p < 0.05, **p < 0.01, ***p < 0.001; ****p < 0.0001 (unpaired *t*-test), which applies to all subsequent statistical analysis

A single seipin ortholog, SEIP-1, was identified in the *C. elegans* proteome by BLAST. Membrane topology predictions suggested that, similar to human seipin, SEIP-1 has two transmembrane helices flanking a conserved central loop region, which may reside in the ER lumen (Supplementary Fig. 1a)^38,39^. To elucidate the function of SEIP-1 in *C. elegans*, we examined *seip-1(tm4221)* mutant animals, which harbored a deletion that removed exon 4 and part of exon 3 of the R01B10.6 open reading frame (Fig. 1a). We could not detect full-length SEIP-1 by western blotting in these animals (Fig. 1d). Therefore, *tm4221* is likely a molecular null allele. We focused our phenotypic analysis on the intestine, which is the major site of fat storage in *C. elegans*^2^. Using label-free stimulated Raman scattering (SRS)^40^, we did not detect significant difference in neutral fat storage between *seip-1* mutants and wild-type animals (Fig. 1b). The functionality of the SRS system was validated for its ability to detect elevated fat storage in *daf-22* mutant animals, which are known to store more triglycerides^41^. We extended our analysis beyond neutral lipids by comparing wild-type and *seip-1* mutant animals with a mass spectrometry-based lipidomic approach^42^ (Supplementary Fig. 1b). The relative amount of two classes of lipids were significantly increased in *seip-1*(−) animals: phosphatidic acid (PA) and diacylglycerol (DAG) (Supplementary Fig. 1c). To investigate how such perturbation in lipid profile correlated with LD morphology, we used mRuby::DGAT-2 as a marker^14^. The median size of LDs in the intestinal cells was reduced from 0.81 μm in diameter in wild-type animals to 0.42 μm in *seip-1*(−) animals (Fig. 1c, e). The alteration of LD size in *seip-1*(−) animals was reproducibly observed when we used DHS-3::GFP as an alternative LD marker (Supplementary Fig. 1e–g)^43^. Taken together, the abundance of small LDs correlated with the over-representation of membrane lipids with small head groups (PA and DAG), possibly because they are more compatible with high negative membrane curvature at junctions between the ER and nascent LDs that fail to expand in the absence of SEIP-1. Nevertheless, not all LDs were reduced in size in *seip-1*(−) animals (Fig. 1e). A similar phenotype of heterogeneous LDs was observed in seipin deficient budding yeast, *Drosophila* cells and mammalian fibroblasts^30–32,34,44^, suggesting that the dependence on seipin for LD homeostasis is evolutionarily conserved.

At the cellular level, energy balance is governed by fat storage in LDs and fat catabolism in peroxisomes and mitochondria. It has been reported that the yeast seipin ortholog Fld1 coordinates LD and peroxisome biogenesis at the ER, in conjunction with Pex30^45^. Interestingly, we observed an increase in the number of intestinal peroxisomes in *seip-1*(−) animals, when we used the peroxisomal thiolase DAF-22 as a marker (Fig. 1f, g). Therefore, SEIP-1 did not appear to be required for peroxisome biogenesis in *C. elegans*. Next, we visualized mitochondria using a red fluorescent protein that is targeted to the outer mitochondrial membrane (TOMM-20(1-54aa):mRuby). We did not detect gross morphological changes in mitochondria in *seip-1*(−) animals (Supplementary Fig. 1h–k). Our results suggest that loss of SEIP-1/seipin function triggers secondary effects on fat catabolism in a species-specific manner.

### SEIP-1 is enriched in peri-LD cages

**Fig. 2:**
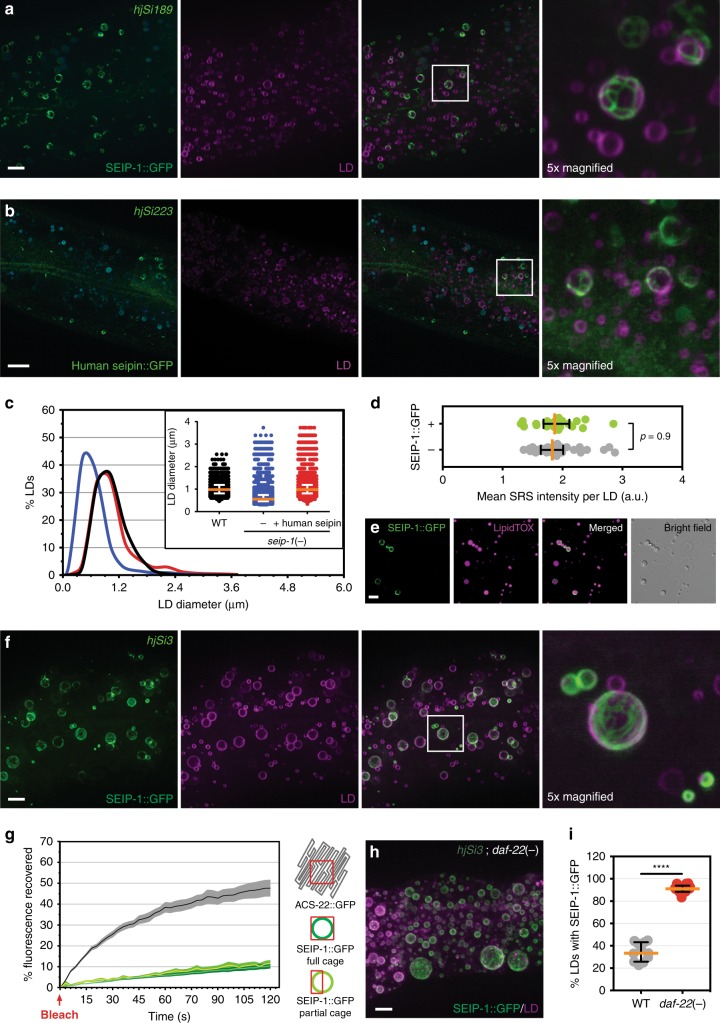
SEIP-1 localizes to peri-LD cages. **a** Visualization of SEIP-1::GFP (*hjSi189*, lane 3 in Fig. 1d) in a larval L4 stage animal. Autofluorescence is pseudocolored blue. The LD marker mRuby::DGAT-2 (*hjSi112*) was used and the mRuby signal is pseudocolored magenta. A projection of 4.5 μm z stack centering at the second intestinal segment is shown. The boxed area was magnified 5× and shown on the right. Scale bar = 5 μm. **b** Visualization of human seipin::GFP (*hjSi223*) in a *seip-1* (*tm4221*) mutant larval L4 stage animal. The human seipin isoform 2 was expressed from a single-copy transgene. A projection of 3 μm z stack centering at the second intestinal segment is shown. The LD marker and autofluorescence signals are displayed as in (**a**). The boxed area was magnified 5× and shown on the right. Scale bar = 10 μm. **c** Frequency distribution of LD diameter in larval L4 stage animals of indicated genotypes. The curve was fitted as in Fig. 1e. Inset: a scatter plot summarizing LD diameter. Total number of LDs measured: WT = 4600; *seip-1*(*tm4221*) = 6069; *seip-1* (*tm4221*);*hjSi223* = 4258. **d** Label-free quantification of lipid concentration of individual LDs by SRS. LDs with a diameter of 0.8–1.7 μm in the second intestinal segment were measured. Total number of LDs analyzed: SEIP-1::GFP (−) = 34; SEIP-1::GFP (+) = 22. **e** Visualization of SEIP-1::GFP in LDs purified from *hjSi3* animals. LDs were stained with LipidTox Deep Red (pseudocolored magenta). **f** As in (**a**), but with an animal overexpressing SEIP-1::GFP (*hjSi3*, lane 4 in Fig. 1d). **g** Percentage of recovered fluorescence of ACS-22::GFP (resident ER protein, *hjSi29*) and SEIP-1::GFP (*hjSi3*) in wild-type animals. The connecting curves show the means and the filled areas show the SEM range. Number of photobleaching events: ACS-22::GFP = 17; SEIP-1::GFP full cage = 15; SEIP-1::GFP partial cage = 20. **h** As in (**f**), but in *daf-22(ok693)* mutant background. **i** The percentage of LDs associated with SEIP-1::GFP in the second intestinal segment in wild-type and *daf-22(ok693)* mutant animals. Number of animals analyzed: wild type (WT) = 11, *daf-22(−)* = 14. For all scatter plots, median with interquartile range is displayed

**Fig. 3:**
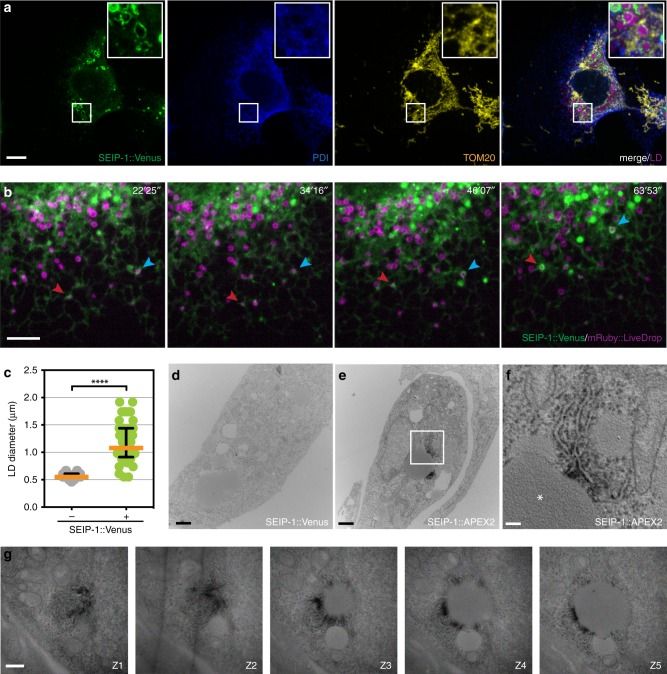
Enrichment in peri-LD cages is an evolutionarily conserved property of seipin. **a** The SEIP-1::Venus protein was stably expressed in COS7 cells. The cells were incubated with oleic acid, fixed and immunostained for endogenous PDI (the ER) and TOM20 (mitochondria). Alexa Fluor (AF)-405 and 594 conjugated secondary antibodies were used for visualizing PDI and TOM20, respectively. AF594 signals were pseudocolored yellow. FAS was used to stain LDs and pseudocolored magenta. A projection of 4.5 μm z stack is shown. The boxed area was magnified 3× and shown in the inset. Scale bar = 10 μm. **b** Time-lapse monitoring of peri-LD cages maturation. The SEIP-1::Venus protein and a nascent LD marker, mRuby::LiveDrop were stably co-expressed in COS7 cells. mRuby signals were pseudocolored magenta. The cells were incubated with oleic acid for 1.5 h before live imaging for another 1 h at 30-s intervals. Arrows with the same color mark the same LiveDrop (+) LDs over time. Scale bar = 5 μm. **c** Quantification of LD size in COS7 cells stably expressing SEIP-1::Venus. A scatter plot of data from 48 LDs of each category, with median and interquartile range is shown. **d** Transmission electron micrograph of a COS7 cell stably expressing SEIP-1::Venus protein. The cell was incubated with oleic acid prior to fixation, and stained with diaminobenzidine (DAB), uranyl acetate, and osmium tetroxide prior to electron microscopy. No dark deposits were detected in this negative control. Scale bar = 1 μm. **e** As in (**d**), but with SEIP-1::V5-APEX2 fusion protein stably expressed. **f** The boxed area in (**e**) was magnified 5×. Dark deposits indicate the localization of SEIP-1::V5-APEX2 at ER tubules. The LD is indicated by an asterisk. Scale bar = 0.2 μm. **g** As in (**e**), but with five consecutive sections (150-nm thick) shown. Scale bar = 0.5 μm

To investigate how SEIP-1 affects LD size, we examined the sub-cellular localization of SEIP-1 by expressing a SEIP-1::GFP fusion protein at the endogenous level from a single-copy transgene *hjSi189*, which is driven by the ubiquitous *dpy-30* promoter (Fig. 1d). The *hjSi189* transgene increased the median LD diameter of *seip-1*(−) animals to 0.83 µm, which is similar to that in wild-type animals (0.81 µm) (Fig. 1e). We concluded that the SEIP-1::GFP fusion protein was functional. In the *C. elegans* intestine, we observed SEIP-1::GFP exclusively in nanotubes that formed rings and cages around a subset of LDs (Fig. 2a). These nanotubes are most likely derived from ER tubules (Fig. 3e–g, Supplementary Fig. 3e–l). We named the SEIP-1::GFP positive structures peri-lipid droplet (peri-LD) cages. Similar observations were made when SEIP-1::tagRFP was expressed (Supplementary Fig. 2h). Furthermore, we used CRISPR to generate transgenic animals that express SEIP-1 with a 3xFLAG epitope tag at its C-terminus. Immunostaining revealed SEIP-1 positive signals around a subset of LDs (Supplementary Fig. 2a, b). Our results suggest that SEIP-1 exerts its effect at ER subdomains that are in close proximity to LDs.

Next, we expressed the human seipin protein fused to GFP from a single-copy transgene in *C. elegans*. Similar to *C. elegans* SEIP-1, human seipin was found to associate with a subset of LDs in tubular structures (Fig. 2b). The human seipin::GFP fusion protein was functional since it restored the median LD size of *seip-1*(−) animals to wild-type level (Fig. 2c). Taken together, the mechanism by which seipin family members are targeted to peri-LD cages is conserved.

### Overexpression of SEIP-1 increased LD size

**Fig. 4:**
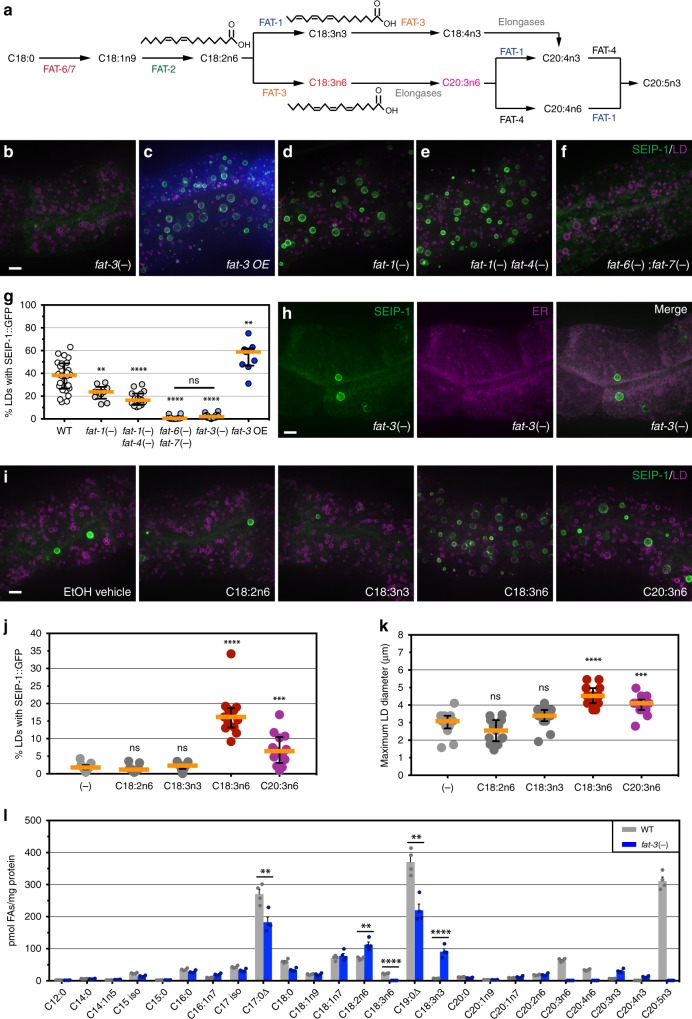
Mislocalization of SEIP-1::GFP in *fat-3* mutant animals. **a** The biosynthetic pathway for polyunsaturated fatty acids in *C. elegans*. **b** Visualization of SEIP-1::GFP (*hjSi3*) in a *fat-3* (*ok1126*) mutant larval L4 stage animal. A projection of 4.5 μm z stack centering at the second intestinal segment is shown. The LD marker mRuby::DGAT-2 (*hjSi112*) was used and the mRuby signal is pseudocolored magenta. **c** As in (**b**), but with a WT animal carrying a transgene that overexpressed FAT-3 (*Ex*[*fat-3p*::*fat-3 genomic DNA*::*SL2*::*tagBFP*]). The presence of blue fluorescence indicates the overexpression of FAT-3. **d**–**f** As in (**b**), but in *fat-1*(*wa9*), *fat-1(wa9) fat-4*(*wa14*), or *fat-6*(*tm331*)*;fat-7*(*wa36*) mutant background. **g** A scatter plot summarizing the percentage of LDs associated with SEIP-1::GFP. Number of animals analyzed: WT = 26, *fat-3(−)* = 18, *fat-1(−)* = 10, *fat-1*(*−*) *fat-4(−)* = 15, *fat-6*(*−*)*;fat-7(−)* = 12, *Ex*[*fat-3*(*+*)] = 9. **h** As in (**b**), but with a luminal ER mCherry marker (*hjSi158*) in a *fat-3 (ok1126)* mutant animal. The mCherry signal is pseudocolored magenta. **i** Visualization of SEIP-1::GFP (*hjSi3*) and mRuby::DGAT-2 (*hjSi112*) in a *fat-3*(*ok1126*) mutant larval L4 stage animals grown in the presence of EtOH vehicle, linoleic acid (LA, C18:2n6), α-linolenic acid (ALA, C18:3n3), γ-linolenic acid (GLA, C18:3n6) or dihomo-γ-linolenic acid (DGLA, C20:3n6) supplementation. A projection of 4.5 μm z stack centering at the second intestinal segment is shown. **j** The effects of PUFAs supplementation on the percentage of LDs associated with SEIP-1::GFP. Number of animals analyzed: (−) = 13, C18:2n6 = 13, C18:3n3 = 14, C18:3n6 = 13, C20:3n6 = 13 (also applied to (**k**)). **k** As in (**j**), but the effects of PUFAs supplementation on the maximum LD diameter is shown. **l** The abundance of all fatty acids detected in WT or *fat-3 (ok1126)* larval L4 stage animals. Mean ± SEM from four independent samples is shown. For all fluorescent images, scale bar = 5 μm. For all scatter plots, median with interquartile range is displayed

The seipin orthologs have been found at ER-LD junctions in mitotic cells^29,31,32^. It has been proposed that the enrichment of seipin at these junctions promote the maturation of nascent LDs^34^. Our observations in *C. elegans* differentiated intestinal cells revealed sustained engagement of SEIP-1(+) structures with a subset of mature LDs. To probe the functional significance of such association, we generated a single-copy transgene (*hjSi3*) that overexpressed a SEIP-1::GFP fusion protein specifically in the *C. elegans* intestine (Fig. 1d). The localization of SEIP-1::GFP to peri-LD cages persisted in these animals. Using mRuby::DGAT-2 to mark all LDs, we determined that ~40% of LDs were surrounded by peri-LD cages in each intestinal cell (Figs. 2f and 4g).

Opposite to the effect of loss of SEIP-1 activity on LD size, there was a significant increase in the median LD size in animals overexpressing SEIP-1::GFP (Fig. 1e). This was in part due to the appearance of LDs > 2 μm in diameter, which were invariably associated with peri-LD cages. Nevertheless, SEIP-1 positive and negative LDs have similar concentration of neutral lipids, as determined by SRS (Fig. 2d). Taken together, we conclude that overexpression of SEIP-1::GFP protein conferred a gain-of-function phenotype: expansion of a subset of LDs.

Next, we purified LDs from *hjSi3* transgenic animals by differential centrifugation and found that peri-LD cages labeled by SEIP-1::GFP remained stably associated with LDs (Fig. 2e). To further examine the spatial relationship between peri-LD cages with the ER tubular network, we generated animals that carry a second single-copy transgene that allowed the visualization of the ER lumen (signal sequence::mCherry::HDEL; *hjSi158*). Fluorescence signals from SEIP-1::GFP at the periphery of peri-LD cages juxtaposed with that from mCherry in the ER lumen (Supplementary Fig. 2i). However, the mobility of SEIP-1 was clearly different from a reference resident ER membrane protein, ACS-22/acyl-coA synthetase^14^. Little recovery was observed when SEIP-1::GFP signals in whole or in part of cages were subjected to photobleaching (Fig. 2g). This was in contrast to the rapid recovery of ACS-22::GFP in the general ER network. Our biochemical and imaging results suggest that peri-LD cages are ER subdomains that preferentially associate with LDs. In addition, SEIP-1 is relatively immobile once incorporated into peri-LD cages, which may in part be due to regional differences of ER membrane constituents.

### Peri-LD cages are ER subdomains

We sought to extend our observations by heterologous expression of SEIP-1 in mammalian cells. To this end, we generated COS7 cell lines that stably expressed a SEIP-1::Venus green fluorescent fusion protein at low levels, using the Sleeping Beauty transposon system^46^. Using endogenous protein disulfide isomerase (PDI) and TOM20 as ER and mitochondrial markers, respectively, we observed SEIP-1::Venus in the ER and bright puncta that were diffraction-limited under basal conditions (Supplementary Fig. 3a). Addition of oleic acid, which promotes LD expansion, caused SEIP-1::Venus enrichment in structures that resembled peri-LD cages that were similar to those observed in *C. elegans* (Fig. 3a). To determine the temporal relationship of SEIP-1 enrichment and LD biogenesis, we generated additional COS7 cell lines that stably expressed low levels of SEIP-1::Venus and the LD biogenesis marker, LiveDrop^34^. Time-lapse microscopy revealed the coincidence of SEIP-1::Venus and LiveDrop signals at puncta that progressively developed into LDs upon oleic acid loading (Fig. 3b, Supplementary Video 1). Finally, LDs marked by SEIP-1::Venus were significantly larger than those that were not marked in the same cells (Fig. 3c). Taken together, our results support a model that selective association of SEIP-1 with nascent LDs not only promotes their initial emergence from the ER but their subsequent expansion.

To study SEIP-1 localization by electron microscopy, we generated additional COS7 cell lines that stably expressed a SEIP-1::APEX2 fusion protein at low levels^47^. The SEIP-1::APEX2 fusion protein could be detected around enlarged LDs, suggesting the function of SEIP-1 was preserved (Supplementary Fig. 3b–d). The APEX2 peroxidase reacts with diaminobenzidine (DAB) to generate local deposits that can be stained with uranyl acetate and osmium tetroxide. Accordingly, we found that SEIP-1::APEX2 was highly enriched on ER tubules in the proximity of LDs in oleic acid loaded cells (Fig. 3e–g, Supplementary Fig. 3e–l). The APEX2 mediated dark deposits were not detected in negative control cells that expressed SEIP-1::Venus (Fig. 3d). The SEIP-1::APEX2 positive ER tubules showed similar morphology to those that are enriched with enzymes in the TAG biosynthetic pathways in *Drosophila* cells^11^. It has been proposed that such ER tubules constitute direct connections between ER and LDs. Our results suggest that peri-LD cages are conserved ER subdomains that support LD expansion.

### Enrichment of SEIP-1 in peri-LD cages required polyunsaturated fatty acids

Is SEIP-1 enrichment in peri-LD cages sensitive to cellular metabolic activities? To address this question, we perturbed mitochondrial fission or fusion by RNAi against *drp-1*/dynamin or *fzo-1*/mitofusin^48,49^, respectively, and observed no significant effect on SEIP-1 targeting (Supplementary Fig. 2d–g). However, attenuation of peroxisomal fatty acid β-oxidation in *daf-22* mutant animals caused promiscuous association of SEIP-1(+) peri-LD cages to almost all LDs (Fig. 2h, i). Our results support a model that the accumulation of fatty acids or their derivatives in *daf-22* mutant animals promotes SEIP-1 enrichment in peri-LD cages, which in turn facilitates the continual expansion of LDs in these animals^41^.

To further elucidate the molecular mechanism for SEIP-1 enrichment in peri-LD cages, we mutagenized *hjSi3* animals with the chemical mutagen ethyl methane sulfonate (EMS). From this forward genetic screen, we obtained two mutants (*hj55* and *hj56*) in which SEIP-1::GFP was largely retained in the ER. Molecular cloning of these mutants identified mutations in the *fat-3* gene, which encodes a fatty acid desaturase for the synthesis of polyunsaturated fatty acids (PUFAs) in *C. elegans* (Fig. 4a)^50^. Specifically, FAT-3 introduces a double bond to C18:2n6 and C18:3n3 to yield C18:3n6 and C18:4n3, respectively. We noted that aberrant localization of SEIP-1::GFP was also observed in mutants that harbored a deletion in the *fat-3* gene (*ok1126*) (Fig. 4b, h). This was not due to a change in the expression level of endogenous SEIP-1 and SEIP-1::GFP (Supplementary Fig. 4a), nor an overt change in LD size distribution of *fat-3*(−) animals (Supplementary Fig. 4f). We concluded that proper SEIP-1::GFP targeting is dependent on PUFAs that are direct or indirect products of the FAT-3 desaturase. Since FAT-3 does not co-localize with SEIP-1 and is distributed throughout the ER network (Supplementary Fig. 4i, j), it is plausible that additional mechanisms may be used to concentrate FAT-3 products in ER subdomains.

Based on previous reports, FAT-3 is required for the biosynthesis of multiple PUFAs^50^. This was confirmed using a quantitative gas chromatography-mass spectrometry (GC-MS) based assay (Fig. 4l). Does SEIP-1::GFP enrichment in peri-LD cages require specific species of PUFAs? To address this question, we examined the localization of SEIP-1::GFP in animals that were deficient in other desaturases of the PUFAs biosynthetic pathway. FAT-1 and FAT-4 encode fatty acid desaturases that act upstream and downstream of FAT-3 (Fig. 4a). The percentage of LDs associated with SEIP-1::GFP was significantly reduced in *fat-1* and *fat-1 fat-4* mutant animals although the effect was not as pronounced as in *fat-3* animals (Fig. 4d, e, g). Again, no change in the expression level of endogenous SEIP-1 and SEIP-1::GFP was observed (Supplementary Fig. 4a). We further considered three desaturases that produce FAT-3 substrates: FAT-2, FAT-6, and FAT-7. Intestinal specific RNAi against *fat-2*, *fat-6*, or *fat-*7 significantly reduced the number of LDs that were associated with SEIP-1::GFP (Supplementary Fig. 4g, h). RNAi against *fat-6* caused simultaneous reduction of *fat-6* and *fat-7* expression levels (Supplementary Fig. 6k). Accordingly, SEIP-1::GFP localization was disrupted partially in *fat-6* or *fat-7* single mutant animals (Supplementary Fig. 4c–e), but completely in *fat-6; fat-7* double mutant animals (Fig. 4f, g). The latter animals also have a higher frequency of small (0.6–0.9 μm) LDs than wild type (Supplementary Fig. 4f). We could not study SEIP-1::GFP localization of *fat-2* mutant animals because of their larval arrest phenotype^50^. When FAT-3 was overexpressed, the percentage of LDs associated with SEIP-1::GFP was increased, suggesting that FAT-3 products play an instructive role in SEIP-1 targeting (Fig. 4c, g). Is a specific FAT-3 product required? To this end, we supplemented FAT-3 substrates (C18:2n6 and C18:3n3) or products (C18:3n6 and C20:3n6) to the *E. coli* diet of *fat-3*(−) animals. The enrichment of SEIP-1::GFP in peri-LD cages was significantly increased by C18:3n6 and C20:3n6 supplementation, while C18:2n6 or C18:3n3 had no effect (Fig. 4i, j). This was accompanied by an increase in the maximal LD diameter in C18:3n6 and C20:3n6 treated animals (Fig. 4k), consistent with the role of SEIP-1 in promoting LD expansion. Taken together, our genetic and dietary supplementation experiments indicate that proper targeting of SEIP-1::GFP is at least partially dependent on specific PUFAs.

### Enrichment of SEIP-1 in peri-LD cages required microbial fatty acids

**Fig. 5:**
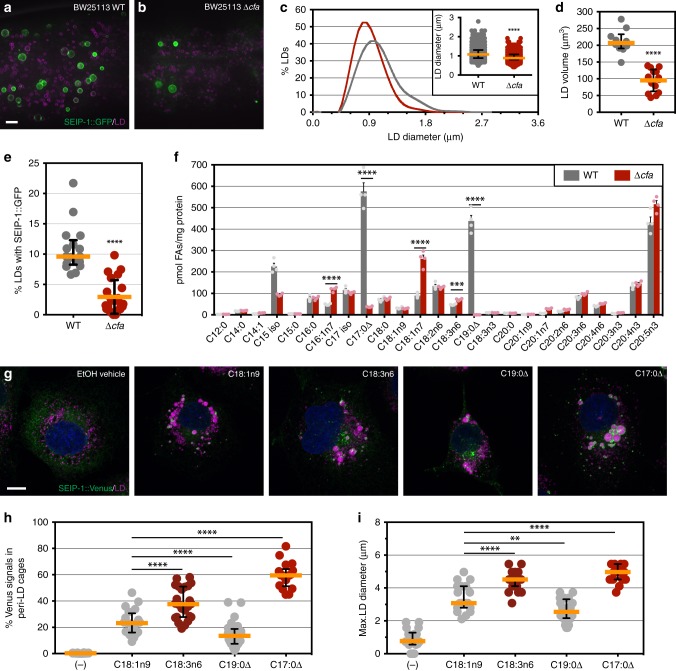
Bacterial cyclopropane-FAs regulate SEIP-1::GFP targeting. **a** Visualization of SEIP-1::GFP (*hjSi3*) in a larval L4 stage animal fed WT *E. coli BW25113*. A projection of 4.5 μm z stack centering at the second intestinal segment is shown. The LD marker mRuby::DGAT-2 (*hjSi112*) was used and the mRuby signal is pseudocolored magenta. Scale bar = 5 μm. **b** As in (**a**), but with an animal fed *Δcfa* mutant *E. coli BW25113*. **c** Frequency distribution of LD diameter in larval L4 stage animals that were fed wild-type (gray) or *Δcfa* (red) *E. coli*. The curve was fitted as in Fig. 1e. Inset: a scatter plot summarizing LD diameter. Total number of LDs measured: *BW25113* WT fed = 2414; *BW25113 Δcfa* fed = 2704. **d** A scatter plot summarizing the total LD volume in the second intestinal segment of each animal. Number of animals analyzed: WT = 10, *Δcfa* *=* 15. **e** The lack of dietary CFA reduced the percentage of LDs associated with SEIP-1::GFP. Number of animals analyzed: WT = 17, *Δcfa* *=* 22. **f** The abundance of all fatty acids detected in worms fed wild-type (gray) or *Δcfa* (red) *E. coli*. Mean ± SEM from four independent samples is shown. **g** COS7 cells stably expressing SEIP-1::Venus were supplemented with either EtOH vehicle, oleic acid (OA, C18:1n9), γ-linolenic acid (GLA, C18:3n6), 11,12-methylene octadecanoic acid (C19:0Δ), or 9,10-methylene hexadecanoic acid (C17:0Δ). Cells were incubated with 100 μM FAs for 24 h. LDs were stained with LipidTox Red (pseudocolored magenta) and nuclei were stained with Hoechst 33342 (blue). A projection of 4.5 μm z stack is shown. Scale bar = 10 μm. **h** The effect of FA supplementation on the percentage of SEIP-1::Venus signals at peri-LD cages. Number of cells analyzed: (−) = 18, C18:1n9 = 20, C18:3n6 = 24, C19:0Δ = 24, C17:0Δ = 18 (also applied to (**i**)). **i** As in (**h**), but the effect of FA supplementation on the maximum LD diameter is shown. For all scatter plots, median with interquartile range is displayed

Our quantitative GC-MS analysis revealed that in addition to a reduction of PUFAs, the abundance of two cyclopropane fatty acids (CFAs), C17:0Δ and C19:0Δ, was also significantly reduced in *fat-3*(−) animals (Fig. 4l). Since CFAs are strictly derived from *E. coli* and are not synthesized by *C. elegans*, our results implied that *fat-3*(−) animals are partially defective in absorbing dietary microbial fatty acids. CFAs are synthesized by the CFA synthase from monounsaturated fatty acids (Supplementary Fig. 5a)^51^. They are major constituents of bacterial phospholipids that are important for membrane integrity under stress^52^. We next asked if the mislocalization of SEIP-1::GFP in *fat-3*(−) animals was in part due to a reduction of diet-derived CFAs. To this end, we obtained the *E. coli* CFA synthase deletion mutant and its parental strain BW25113 from the Keio collection^53^. Using GC-MS, we confirmed that the *Δcfa* strain indeed was unable to synthesize C17:0Δ and C19:0Δ (Supplementary Fig. 5b). Next, we fed *hjSi3; hjSi112* transgenic animals that expressed SEIP-1::GFP and mRuby::DGAT-2 with BW25113 and *Δcfa E. coli*. Using mRuby::DGAT-2 as a pan-LD marker in the intestine, we noted a reduction of median LD diameter and total LD volume in animals fed *Δcfa E. coli* (Fig. 5c, d). Furthermore, the percentage of LDs that are associated with SEIP-1::GFP was also significantly reduced (Fig. 5e). Quantitative GC-MS analysis indicated an almost complete loss of C17:0Δ and C19:0Δ, and a concomitant increase of C16:1n7 and C18:1n7 in the fatty acid profile of worms fed *Δcfa E. coli* (Fig. 5f). This was consistent with C16:1 and C18:1n7 being the precursors of C17:0Δ and C19:0Δ in *E. coli*, respectively (Supplementary Fig. 4a, b). Based on our model, residual targeting of SEIP-1::GFP to peri-LD cages in *fat-3*(−) animals could be attributed to reduced, but measurable levels of CFAs. Indeed, complete disruption of SEIP-1::GFP targeting ensued when *fat-3*(−) animals were fed *Δcfa E. coli* (Supplementary Fig. 5c). Our results demonstrate that *C. elegans* obtain CFAs from their microbial diet and incorporate them efficiently into their cellular lipids. Such dietary CFAs have a significant impact on fat content, LD morphology and SEIP-1 targeting.

### PUFAs and CFAs regulate SEIP-1 targeting in mammalian cells

To test the effect of PUFAs and CFAs on SEIP-1 targeting in a simplified, heterologous system, we used the previously generated COS7 cell lines that stably expressed a SEIP-1::Venus fusion protein at low levels. In comparison with oleic acid (C18:1n9), supplementation of C18:3n6 and C17:0Δ, but not C19:0Δ had significantly stronger effects on promoting LD expansion, which correlated with SEIP-1::Venus enrichment in peri-LD cages (Fig. 5g–i, Supplementary Fig. 5g, h). Furthermore, the effect of C17:0Δ was dose-dependent (Supplementary Fig. 5d–f). Our results suggest that polyunsaturated C18:3n6 and microbial C17:0Δ play conserved roles in regulating SEIP-1 targeting in mammalian cells.

### Attenuation of phosphatidylcholine synthesis enhances SEIP-1 targeting to peri-LD cages

**Fig. 6:**
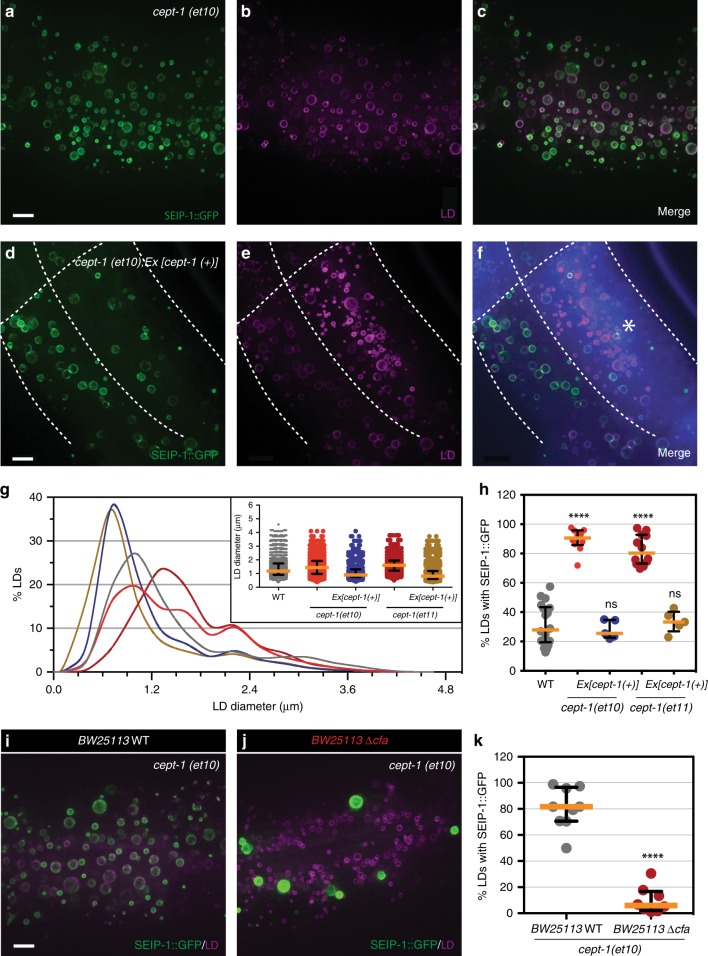
Attenuation of phosphatidylcholine synthesis promotes SEIP-1::GFP targeting to peri-LD cages. **a** Visualization of SEIP-1::GFP (*hjSi3*) in a *cept-1*(*et10*) mutant larval L4 stage animal. A projection of 4.5 μm z stack centering at the second intestinal segment is shown. **b** As in (**a**), but with a mRuby::DGAT-2 LD marker (*hjSi112*). The mRuby signal is pseudocolored magenta. **c** As in (**a**), but with GFP and mRuby signals merged. **d** As in (**a**), but with a *cept-1*(*et10*) mutant animal carrying a rescuing transgene (*Ex*[*vha-6p*::*cept-1a cDNA*::*SL2*::*tagBFP*]). The polycistronic transgene enabled the co-expression of CEPT-1 and tagBFP. The white dotted lines approximate the cell boundaries. **e** As in (**d**), but with a mRuby::DGAT-2 LD marker (*hjSi112*). The mRuby signal is pseudocolored magenta. **f** As in (**d**), but with GFP, mRuby and tagBFP signals merged. Expression of CEPT-1, as indicated by blue fluorescence in the cell marked with an asterisk, restored differential targeting of SEIP-1::GFP to peri-LD cages surrounding a subset of LDs. **g** Frequency distribution of LD diameter in larval L4 stage animals with indicated genetic backgrounds. The curve was fitted as in Fig. 1e. Inset: a scatter plot summarizing LD diameter. Total number of LDs measured: WT = 2347; *cept-1*(*et10*) *=* 1306; *cept-1*(*et10*)*;Ex[cept-1*(*+*)] = 854; *cept-1*(*et11*) = 1319; *cept-1*(*et11*);*Ex*[*cept-1*(*+*)] *=* 936. **h** The percentage of LDs associated with SEIP-1::GFP. Number of animals analyzed: WT = 18, *cept-1*(*et10*) = 10, *cept-1*(*et10*)*;Ex*[*cept-1*(*+*)] = 5, *cept-1*(*et11*) = 12, *cept-1*(*et11*);*Ex[cept-1*(*+*)] = 5. **i** As in (**a**), but fed with WT *E. coli BW25113*. **j** As in (**a**), but fed with *Δcfa* mutant *E. coli BW25113*. **k** The percentage of LDs associated with SEIP-1::GFP in *cept-1*(*et10*) animals fed different *E. coli* diets. Number of animals analyzed: WT = 9, *Δcfa* *=* 8. For all fluorescent images, scale bar = 5 μm. For all scatter plots, median with interquartile range is displayed

Phospholipids with PUFAs and CFAs as acyl chains may shape the membrane micro-environment of the ER. Therefore, we used an intestine-specific RNAi strain to knockdown a number of genes that encode phospholipid biosynthetic enzymes in *C. elegans*, in order to identify cell-autonomous, membrane-structural regulators of SEIP-1 targeting. We found that intestine-specific depletion of *pcyt-1* and *cept-1* but not *cept-2* caused promiscuous association of SEIP-1(+) peri-LD cages with almost all LDs (Supplementary Fig. 6a). PCYT-1 and CEPT-1 act sequentially in the Kennedy pathway for phosphatidylcholine (PC) synthesis in *C. elegans*^54^, suggesting that SEIP-1 targeting is sensitive to cellular PC content. Our RNAi results could be recapitulated in mutant animals that carried two different *cept-1* loss-of-function alleles^55^ (Fig. 6a–c, h). These animals also showed an increase in the median LD size (Fig. 6g). Overexpression of CEPT-1 restored SEIP-1 targeting to peri-LD cages around a subset of LDs and reduced the median LD size of these mutant animals (Fig. 6d–h). Interestingly, the *cept-1* loss-of-function phenotypes could be suppressed by withdrawing dietary CFAs (Fig. 6i–k) or by blocking FAT-3 or FAT-6 mediated PUFAs synthesis (Supplementary Fig. 6e–j). We conclude that the precise control of CFAs, PUFAs, and PC levels is necessary for maintaining SEIP-1 dependent LDs diversity.

### SEIP-1 enrichment in peri-LD cages is partially dependent on COPII

**Fig. 7:**
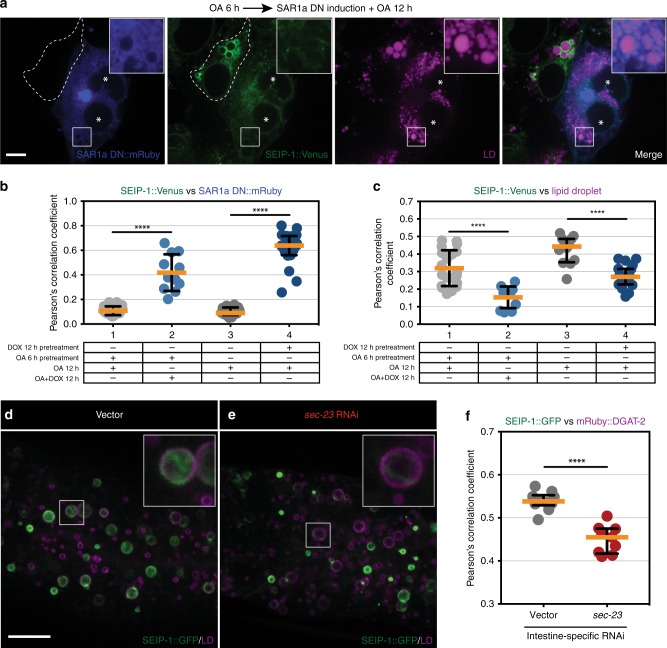
Inhibition of COPII perturbs the proper targeting of SEIP-1. **a** Doxycycline (DOX)-inducible overexpression of SAR1a(H79G)::mRuby (dominant-negative (DN) mutant) in COS7 cells that stably expressed SEIP-1::Venus. Cells were pre-loaded with 400 μM oleic acid (OA) for 6 h and induced by 1 μg/mL DOX for 12 h in the presence of OA. Lipid droplets were stained by FAS and pseudocolored magenta, mRuby signal is pseudocolored blue. Boxed regions were magnified 3× and shown in the inset. Single focal planes are shown. Asterisks mark the cells that overexpressed SAR1a DN mutant, while the adjacent cells with relatively low level of induction are outlined by dashed lines. Scale bar = 10 μm. **b** Colocalization coefficient of SAR1a DN::mRuby and SEIP-1::Venus signals, calculated from 4.5 μm z-stack volumes. Number of cells analyzed: column 1 = 20, column 2 = 11, column 3 = 12, column 4 = 19 (also applied to (**c**)). Columns 1 and 3 serve as controls to columns 2 and 4, respectively. Images in (**a**) were quantified and summarized in column 2; images in Supplementary Fig. 7b were quantified and summarized in column 4. **c** As in (**b**), but with the colocalization coefficient of SEIP-1::Venus and FAS (LD dye) signals calculated. **d** Visualization of SEIP-1::GFP (*hjSi3*) with control RNAi knockdown in a larval L4 stage animal. A LD marker mRuby::DGAT-2 (*hjSi112*) was used and the mRuby signal is pseudocolored magenta. Projections of 4.5 μm z stack centering at the second intestinal segment are shown. The boxed region is magnified 3× and shown in the inset. Scale bar = 10 μm. **e** As in (**d**), but with intestine-specific RNAi knockdown of *sec-23*. **f** Colocalization coefficient of SEIP-1::GFP and mRuby::DGAT-2 signals calculated from 4.5 μm z-stacked volumes in the second intestinal segment. Number of animals analyzed: vector = 10, *sec-23* = 9. For all scatter plots, median with interquartile range is displayed

SEIP-1 is an integral membrane protein. We therefore asked if the canonical secretory pathway is involved in the enrichment of newly synthesized SEIP-1 in peri-LD cages. To this end, we used the Sleeping Beauty transposon system to generate COS7 cell lines that constitutively expressed low levels of SEIP-1::Venus. In addition, these cells could be induced to express a GTP-locked, dominant negative (DN) form of SAR1a(H79G)::mRuby by doxycycline (DOX). The expression of SAR1a(DN)::mRuby effectively retarded COPII-dependent membrane trafficking, without altering the SEIP-1::Venus expression level (Supplementary Fig. 7a). However, SEIP-1::Venus was not found in peri-LD cages upon SAR1a(DN) induction (Fig. 7a and Supplementary Fig. 7b, c). Accordingly, we observed significantly enhanced colocalization of SEIP-1::Venus and SAR1a(DN)::mRuby signals, indicating the retention of SEIP-1 to the ER (Fig. 7b). This was accompanied by a decrease in the colocalization of SEIP-1::Venus with signals from the fluorescent LD dye, FAS^56^ (Fig. 7c). Time-lapse microscopy indicated that SEIP-1::Venus puncta failed to elaborate into peri-LD cages upon oleic acid loading, when SAR1a(DN) was induced (Supplementary Fig. 7d, Supplementary Video 2). In *C. elegans*, the targeting of SEIP-1 to peri-LD cages was partially impaired when *sec-23*, which encodes an ER exit site component, was knocked down by RNAi in the intestine (Fig. 7d–f). It has been shown that COPII-decorated ER exit sites (ERES) are in close proximity of LDs, for the delivery of specific LD proteins^57^. It is plausible that COPII plays a related role in SEIP-1 targeting.

## Discussion

In this paper, we combined genetic, biochemical, light and electron microscopy approaches to demonstrate the preferential enrichment of SEIP-1/seipin to an ER subdomain, termed the peri-LD cage. Peri-LD cages selectively engage a subset of nascent LDs and promote their subsequent expansion. Recruitment of SEIP-1 to peri-LD cages demands PUFAs and microbial CFAs. More specifically, γ-linolenic acid (C18:3n6) and cyclopropane fatty acid (C17:0Δ) have the strongest effect on SEIP-1 targeting in *C. elegans* and mammalian cells. Our results suggest that the heterogeneity of LDs can be enforced not only by LD proteins, but through inter-organelle contacts with the ER.

How does the enrichment of SEIP-1 in peri-LD cages increase LD size? In the energy-replete state, a balance of lipolysis and TAG synthesis determines LD size^11,58^. Attenuation of lipolysis, i.e., blocking the release of fatty acids from triglycerides in LDs, will therefore increase LD size. Lipolysis is rate-limited by the recruitment of adipose triglyceride lipase (ATGL) to the LD surface^59^. We have previously shown that the *C. elegans* ATGL ortholog (ATGL-1) is found at LDs^41^. Since the ATGL-1::GFP fusion protein was observed at the surface of LDs with or without peri-LD cages (Supplementary Fig. 2j), we concluded that peri-LD cages did not impede lipolysis by blocking ATGL-1 recruitment. Development of assays for measuring the lipolytic rate at the single LD level will be necessary to probe additional effects of peri-LD cages on lipolysis.

The de novo synthesis of triglycerides (TAG) is dependent on LD associated DGAT2. In mammalian cells, DGAT2 transits from the ER to the LD surface upon lipid loading^11,19^. In *C. elegans*, the DGAT2 ortholog (DGAT-2) associates stably with LDs^14^. Throughout this study, mRuby::DGAT-2 was used to mark all LDs and we did not observe enhanced DGAT-2 residency at LDs with peri-LD cages. Nevertheless, it is plausible that peri-LD cages can increase the local concentration of additional proteins that are rate-limiting for TAG synthesis. These proteins should be well conserved since peri-LD cage association increased LD size in both *C. elegans* and mammalian cells. For example, the glycerol-3-phosphate acyltransferase 4 (GPAT4), which acts upstream of DGAT2 in the TAG synthesis pathway, is also known to migrate from the ER to LDs and functionally couple with seipin^11,60^. Although the mammalian GPAT4 shares sequence homology with a number of *C. elegans* acyltransferases, its functional ortholog awaits to be identified definitively.

In budding yeast, several proteins, including Ldb16 and Ldo45, act with the seipin ortholog Fld1 at ER-LD junctions to regulate LD size^61–66^. In addition, human Promethin/TMEM159, a remote ortholog of Ldo45, has been shown to associate with LDs^67,68^. However, BLAST homology searches for their orthologs in *C. elegans* did not return significant hits. Since the conserved central loop region of seipin orthologs is flanked by divergent N- and C-termini, it is tempting to speculate that seipin orthologs may have conserved and species-specific partners that modulate LD morphology in a convergent manner.

Cellular membranes are in large part composed of phospholipids that consist of a head group and two fatty acyl chains. The combination of saturated, monounsaturated, and polyunsaturated acyl chains in phospholipids modulates the packing and curvature of membranes that contribute to organelle identity and function^36,69^. Although humans obtain PUFAs from their diet, PUFAs are synthesized endogenously by a set of desaturases in *C. elegans*^50^. Based on biochemical and molecular dynamics simulations, PUFAs have the unique ability to support the formation of highly curved and densely packed membranes^70^. It is plausible that ER tubules that constitute peri-LD cages are composed of such membranes since SEIP-1 was immobile once incorporated into peri-LD cages (Fig. 2g). This could be due to the tight fitting of fatty acyl chains around SEIP-1. PUFAs in peri-LD cages may also act as diffusion barriers to additional proteins, such as those that support LD expansion. Our work supports the idea that PUFAs have fundamental structural roles in defining membrane territories.

It has been reported that Fld1/seipin and Pex30 define an ER subdomain for LD and peroxisome biogenesis in yeast^45,71^. Therefore, we were intrigued by the possibility that Pex30 could be used to label SEIP-1(+) peri-LD cages in heterologous systems. To this end, we generated COS7 cell lines that stably co-expressed SEIP-1::Venus and Pex30::mRuby. Under basal conditions, no significant colocalization of the two proteins was observed (Supplementary Fig. 8a). However, induction of LD expansion by oleic acid loading prompted the co-enrichment of SEIP-1 and Pex30 in peri-LD cages (Supplementary Fig. 8b). Interestingly, time-lapse microscopy revealed that the formation of SEIP-1::Venus puncta preceded Pex30::mRuby puncta at the initial phase of oleic acid loading (Supplementary Fig. 8c, Supplementary Video 3). It is tempting to speculate that the sequential enrichment of SEIP-1 and Pex30 reflects stepwise assembly of additional lipid or protein components of peri-LD cages.

There is an increasing appreciation on how the host metabolism can be modulated by the microbiome, in part through microbial metabolites^72,73^. Our work reveals cyclopropane fatty acids (CFAs, C17:0Δ, and C19:0Δ) as new players in host-microbe interaction. Cyclopropane fatty acyl chains are generated through the action of CFA synthase on monounsaturated precursors in membrane phospholipids of *E. coli* and other bacteria^51^. Since there is no eukaryotic CFA synthase, the detection of CFAs in *C. elegans* indicates their effective absorption and incorporation into host cellular lipids. CFAs have also been detected in human adipose tissue where seipin is strongly expressed^22,37^. Nevertheless, it was hitherto unclear how CFAs play a role in host metabolism. Our work hinted at one possible mechanism: CFA-dependent protein targeting.

In summary, our results reveal unexpected and conserved roles of microbial and polyunsaturated fatty acids on cellular fat storage. They modulate SEIP-1/seipin targeting to peri-LD cages and in turn promote LD expansion in *C. elegans* and mammalian cells. The selective association of peri-LD cages to a subset of LDs also strengthen the notion of LD heterogeneity^9^. Distinct populations of LDs may carry specific cargos beyond neutral lipids. They may be subject to differential turnover during fed or fasted states. Our results implicate the selective deployment of dietary and endogenous fatty acids in defining organelle identity through specialized membrane environments.

## Methods

### Strains and transgenes

The wild-type *C. elegans* strain was Bristol N2. All experimental animals were maintained at 20 °C. The following alleles were obtained from the *Caenorhabditis* Genetics Center (CGC), which is funded by NIH Office of Research Infrastructure Programs (P40 OD010440): LG III, *unc-119(ed3)*; LG X, *cept-1*(*et10*)*, cept-1*(*et11*); LG IV, *fat-1 fat-4*(*wa9 wa14*)*, fat-3*(*ok1126*); LG V, *sid-1*(*qt78*). The *seip-1*(*tm4221*) allele was obtained from Dr. Shohei Mitani (National Bioresource Project for the nematode). The following transgenes or CRISPR-generated alleles were used: *hjSi3*[*vha-6p*::*seip-1 cDNA*::*GFP_TEV_3xFLAG*::*let-858 3*′*UTR*] *II, hjSi56*[*vha-6p*::*3xFLAG_TEV_GFP*::*F59A1.10 coding*::*let-858 3*′*UTR*] *IV, hjSi112*[*vha-6p*::*3xFLAG_TEV_mRuby2*::*dgat-2 cDNA*::*let-858 3*′*UTR*] *IV, hjSi127*[*vha-6p*::*mRuby2*::*CB5*::*let-858 3*′*UTR*] *IV, hjSi158*[*vha-6p*::*SEL-1(1-79aa)*::*mCherry*::*HDEL*::*let-858 3*′*UTR*] *I, hjSi189*[*dpy-30p*::*seip-1 cDNA*::*GFP_TEV_3xFLAG*::*tbb-2 3*′*UTR*] *II, hjSi222*[*fat-3p*::*fat-3 genomic DNA*::*GFP-TEV-3xFLAG*::*fat-3 3*′*UTR*] *I, hjSi223*[*dpy-30p*::*human seipin isoform 2 cDNA (codon-optimized)*::*GFP_TEV_3xFLAG*::*tbb-2 3*′*UTR*] *II, hjSi434*[*dpy-30p*::*seip-1 cDNA*::*tagRFP*::*tbb-2 3*′*UTR*] *II, hjSi489*[*dpy-30p*::*tomm-20(1-54aa)::mRuby*] *II, hjSi494*[*vha-6p*::*sid-1 cDNA*::*dhs-28 3*′*UTR*] *I*, *hjEx21, hjEx22*[*vha-6p*::*cept-1a cDNA_SL2_tagBFP2*::*let-858 3*′*UTR*], *hjEx23*[*fat-3p*::*fat-3 genomic DNA_SL2_tagBFP2*::*fat-3 3*′*UTR, dhs-3*(*hj120*) [*dhs-3*::*GFP_3xFLAG], seip-1*(*hj196*) [*seip-1*::*TEV_3xFLAG*]*, daf-22*(*hj234*) [*tag-RFP_3xFLAG*::*daf-22*].

### *C. elegans* genetic screen

To isolate mutant animals with altered localization of SEIP-1::GFP, we mutagenized *hjSi3* animals with ethyl methane sulfonate (EMS) using standard procedures. We screened ~38,000 haploid genomes and selected mutant F2 animals on a UV fluorescence dissecting microscope (Leica). Mutant animals were back-crossed with *hjSi3* animals at least twice and their phenotype was subsequently verified by confocal microscopy. For molecular cloning, we focused on one complementation group comprised of *hj55* and *hj56* alleles. Genetic mapping with the Hawaiian isolate CB4856 placed both alleles on LGIV. Molecular lesions in the *fat-3* gene were identified by whole-genome sequencing (Illumina) and verified by targeted Sanger sequencing. The *hj55* and *hj56* alleles encode the mutations Gln325Stop and Gly329Asp, respectively. The *fat-3(ok1126)* deletion allele, outcrossed four times with N2, was subsequently used as the reference strain. The mislocalization of SEIP-1::GFP in the *hjSi3; fat-3(ok1126)* strain could be rescued by a *fat-3p*::*fat-3 genomic DNA*::*SL2*::*tagRFP*::*fat-3 3*′*UTR* transgene.

### RNA interference-based knockdown in *C. elegans*

RNA interference (RNAi) was performed based on published methods^73^. The RNAi vectors for knocking down *fat-2, fat-6* or *fat-7* were taken from the Ahringer library and transformed into OP50 [*rnc14*::*DTn10 laczgA*::*T7pol camFRT*]. The RNAi vectors for knocking down *drp-1, fzo-1, pcyt-1, cept-1,* and *cept-2* were constructed for this study and transformed into the above OP50 strain. Primers used for molecular cloning: *drp-1*, 5′-TCAACAGAATATTCTCGACAACAAAC-3′ and 5′-TTCAAATGAGACTTCTGGTACG-3′; *fzo-1*, 5′-CTTTCTCCAGAATTCGATAGC-3′ and 5′-ATACAATGCTCAAAATGTCG-3′; *pcyt-1*, 5′-CTGTAACTTACAGCTGGCCG-3′ and 5′-CGTTCAAACCTCCGTCCTTATG-3′; *cept-1*, 5′-CCAGTCTTCGTCACATTGAAC-3′ and 5′-CGTCGCAATCCATAACAAGAC-3′; *cept-2*, 5′-GCTACGCTTTGCAATTAGG-3′ and 5′-CAATAAATGCCCATATGGTCG-3′. Fresh overnight cultures of OP50 RNAi clones were seeded on NGM plates with 0.4 mM IPTG and 100 μg/mL Ampicillin. The seeded plates were incubated at room temperature for 1 day. Six to eight L4 larval P0 animals of each genotype were picked onto the plates and removed after 2 days. F1s were imaged at the L4 larval stage. To achieve intestine-specific knockdown, we generated a single-copy transgene that expressed wild-type SID-1 protein under the control of intestine-specific *vha-6* promoter (*hjSi494)*. The transgene was then crossed into *sid-1*(*qt78*) mutant animals^74^. *hjSi3* [SEIP-1::GFP] and *hjSi112* [mRuby::DGAT-2] were subsequently introduced into *hjSi494;sid-1(qt78)* and used for RNAi-based knockdown following the above procedures.

### Fluorescence imaging of *C. elegans* and mammalian cells

Fluorescence images of L4 larval animals or mammalian cells were acquired on a spinning disk confocal microscope (AxioObeserver Z1, Carl Zeiss) equipped with a piezo Z stage using a ×100, numerical aperture (NA) 1.46 oil Alpha-Plan-Apochromat objective, on an Neo sCMOS camera (Andor) controlled by the iQ3 software (Andor). For GFP, a 488 nm laser was used for excitation and signals were collected with a 500–550-nm emission filter. For mCherry and tagRFP, a 561 nm laser was used for excitation and signals were collected with a 580.5–653.5-nm emission filter. For autofluorescence from lysosome-related organelles, a 488 nm laser was used for excitation and signals were collected with a 580.5–653.5-nm emission filter. For tagBFP, a 405 nm laser was used for excitation and signals were collected with a 417–477-nm emission filter.

Optical sections were taken at 0.5 μm intervals and z stacks of images were exported from iQ3 to Imaris 8 (Bitplane) for processing and 3D reconstruction. Ten focal planes in total (4.5 μm in *z* axis) were used for all projected fluorescence images unless otherwise indicated. For worm intestine, the diameter of each LD present in the second intestinal segment was all manually fitted using the spot function in Imaris. Pearson correlation coefficients were calculated in Imaris without thresholding.

To quantify enrichment of SEIP-1::Venus signals at peri-LD cages upon FAs loading, FA supplementation was performed blindly (i.e., the identity of FAs loaded to each plate was not known to the experimenter who took the images and analyzed the data). Ten optical sections at 0.5 μm intervals of each cell were exported as raw greyscale images from Imaris and subsequently reconstituted in ImageJ as maximum signal projections. For each projection, a mean background value was initially calculated and used as the threshold. The intensity of Venus signals at all peri-LD cages to that in the overall cell area was calculated using the ROI function in ImageJ and used to assess the enrichment of SEIP-1::Venus at peri-LD cages.

### Maintenance of mammalian cells

COS7 cells (ATCC, CRL-1651) were cultured in DMEM (Gibco) with 10% FBS (Gibco) and 1% antibiotic-antimycotic (Gibco) in humidified incubators flowed with 5% CO_2_.

### FAs supplementation in mammalian cells

Supplementation was performed based on a published method^75^. In short, the 100 mM stock solution of each FAs was diluted immediately into the complete medium pre-heated to 60 °C. Medium was then equilibrated to 37 °C before loading. To induce LD expansion, cells were incubated with 400 μM FAs for 12 h unless otherwise indicated.

### Time-lapse live cell imaging

Cells were initially seeded in 35-mm MatTek glass-bottom dishes coated with poly-D-lysine. Upon OA loading, cells were imaged in a stage-top incubator that was flowed with humidified air with 5% CO_2_, and held at 37 °C by a CU-501 temperature controller (Chamlide TC^TM^). Time-lapse images were acquired using the above confocal system in 30-s intervals controlled by a definitive focus (fc12, Zeiss) unless otherwise indicated.

### Fluorescence recovery after photobleaching (FRAP) assay

Photobleaching was performed on larval L4 stage animals using the spinning disk confocal microscope described above. The region of interest (ROI) was chosen to cover the entire or half peri-LD cage in *hjSi3* animals. For each FRAP event, the size of the ROI was kept constant for both background and reference regions. One or more ROIs of at least eight animals were examined for each strain. Normalized fluorescence intensity (NI) was calculated using the following formula, taking background fluorescence (Back) and fluorescence in an unbleached reference ROI (Ref) into consideration.

1 $$NI_{{\mathrm{ROI}}t1} = \left( {\frac{{I_{{\mathrm{ROI}}t1} - I_{{\mathrm{Back}}t1}}}{{I_{{\mathrm{ROI}}t0} - I_{{\mathrm{Back}}t0}}}} \right) \ast \left( {\frac{{I_{{\mathrm{Ref}}t0} - I_{{\mathrm{Back}}t0}}}{{I_{{\mathrm{Ref}}t1} - I_{{\mathrm{Back}}t1}}}} \right)$$

### Label-free lipid imaging in *C. elegans*

For analyzing single lipid droplets, an integrated femtosecond (fs) stimulated Raman scattering (SRS) and two-photon excited fluorescence (TPEF) microscope system was used for label-free imaging of LDs in *C. elegans*^40^. L4 larval animals (*hjSi3*) were dissected in the physiological buffer (150 mM NaCl, 3 mM KCl, 2 mM MgCl_2_, 3 mM CaCl_2_, 10 mM glucose and 15 mM HEPES; 340–345 mmol/kg) containing 0.2 mM levamisole and mounted on a 2% agarose pad. Raw signals from GFP, SRS and tryptophan were acquired sequentially from a field of view (FOV) of 30 × 30 µm in z stacks of 7 µm at 0.5 µm intervals. For quantification, an averaged background noise ($\overline {I_{{\mathrm{noise}}}}$) from photodiode and lock-in amplifier (LIA) was measured and SRS signals of glycerol (*I*_glycerol_) in the same FOV were acquired for normalization prior to acquisition of the GFP, SRS, and tryptophan signals. LD in dissected *hjSi3* animals that was 0.8–1.7 µm in diameter and present in focal plane 1–8 (3.5 µm in *z* axis) was used for quantification. For each LD in each focal plane, total intensity of normalized SRS signals (*I*_plane_) was calculated by:

2 $$I_{{\mathrm{plane}}} = \mathop {\sum }\limits_{i = 1}^n \frac{{I_{{\mathrm{raw}}\;i} - \overline {I_{{\mathrm{noise}}}} }}{{I_{{\mathrm{glycerol}}\;i} - \overline {I_{{\mathrm{noise}}}} }}$$

where *I*_raw *i*_ is the intensity of *i*th pixel present in the LD and *n* is the number of all pixels enclosed in the LD. *I*_plane_ from all focal planes that each LD spanned were summed up as the total intensity of each LD (*TI*_LDs_). Volume of each LD was calculated using ImageJ in raw SRS images.

For whole-worm lipid measurement, live L4 larval animals were directly immersed in the physiological buffer containing 0.2 mM levamisole and mounted on a 8% agarose pad. For each animal, a projection image with maximal SRS intensity was acquired using a picosecond SRS microscope system as previously developed equipped with a × 20 air objective (Plan-Apochromat, 0.8 NA, Zeiss)^76^. Quantification was performed according to published protocols^77^. Briefly, raw total intestinal SRS intensity of each animal was adjusted by $\overline {I_{{\mathrm{noise}}}}$ and *I*_glycerol_ as described above, and subsequently averaged by the total intestinal pixel number. At least 20 animals were imaged and quantified for each genotype.

### Antibodies and western blotting for *C. elegans*

Antibodies against SEIP-1 were raised by injection of the peptide ^261^KKEEPGLLDLRKRK, corresponding to the C-terminus of SEIP-1, into rabbits (YenZym). Antibodies were purified against the antigen immobilized on the AminoLink Plus Coupling resin (Pierce, #44894) and used in western blotting at a dilution of 1:100. IRDye 800CW donkey anti-rabbit secondary antibodies (LI-COR) were used at 1:5000. Fluorescence signals were visualized using an Odyssey Infrared Imaging system (LI-COR) and analyzed using Odyssey software according to manufacturer’s instructions. Background signals were measured from multiple areas of the blot without proteins, averaged, and subtracted from integrated intensity of each protein band. The non-specific signal from a ~95 kDa protein was used as the loading control unless otherwise indicated. An uncropped scan of the western blot in Fig. 1d is shown in Supplementary Fig. 9.

### LDs isolation from *C. elegans*

LDs isolation from *C. elegans* was performed using a published method^78^. In brief, around 100,000 synchronized L4 larval animals were washed and resuspended in buffer A (20 mM Tricine, 250 mM sucrose, pH 7.8, 0.5 mM PMSF). The worm resuspension was dounced on ice in a 15 mL Tenbroeck tissue grinders (Pyrex) for 60 initial times and subsequently transferred to a 15 mL Potter-Elvehjem tissue grinder (Wheaton) for douncing 80 more times. Worms debris and post-nuclear supernatant (PNS) was removed by centrifuge at 1000 × *g* for 15 min. The resulted supernatant was overlaid with buffer B (20 mM HEPES, 100 mM KCl, 2 mM MgCl_2_, pH 7.4) at equal ratio and used for gradient-ultracentrifugation in SW41Ti swinging bucket rotor (Beckman-Coulter). LDs were collected at buoyant fractions after a 2-h centrifugation at 10,000 × *g*.

For fluorescence imaging, 10 μl LD sample was diluted with 90 μl buffer B and incubated with 1:1000 diluted LipidTox Deep Red (Thermo Fisher, #H34477) for 30 min on ice. Fluorescence images were acquired using a confocal microscope (Olympus, FV1000).

### Dietary supplementation of polyunsaturated fatty acids (PUFAs)

Pure PUFAs: linoleic acid (LA), α-linolenic acid (ALA), γ-linolenic acid (GLA), and dihomo-γ-linolenic acid (DGLA) (Nu-Chek Prep) were dissolved in absolute ethanol at a final concentration of 50 mM as stock solutions. Each stock solution was freshly diluted in sterilized Milli-Q water (30 μl 50 mM PUFAs and 120 μl water) and overlaid on top of a seeded bacteria lawn in a 6 cm nematode growth media (NGM) plate. The plates were immediately dried in a dark laminar flow hood for 30 min. For fluorescence imaging, six *hjSi3;hjSi112* or *hjSi3;hjSi112 ok1126* L4 larvae were transferred onto each PUFAs- supplemented plate and allowed to lay eggs for 2 days. Two replicates were performed for each strain in each PUFAs-supplemented condition. For each replicate, 6–8 of their progenies at the L4 larval stage were imaged.

### Generation of stable mammalian cell lines

COS7 cells stably expressing SEIP-1::Venus or SEIP-1::V5-APEX2 were generated by the Sleeping Beauty (SB) transposon system^46,47^. Cells were co-transfected with pCMV(CAT)T7-SB100 that bore the SB Transposase and a plasmid based on pSBbi-Hyg (SEIP-1::Venus) or pSBbi-RH (SEIP-1::V5-APEX2) using Lipofectamine 2000 (Life Technologies). Selection of stable integrants was facilitated by 0.5 mg/mL hygromycin for at least 7 days. The resulting cell population was further sorted using a Becton Dickinson Influx system and arbitrarily sub-divided into “Low”, “Medium”, and “High” fractions based on the fluorescence intensity of Venus (for SEIP-1::Venus cells) or TdTomato (for SEIP-1::V5- APEX2 cells). The “Low” fractions of all cell lines were used. For COS7 cells that constitutively co-expressed SEIP-1::Venus and mRuby::LiveDrop (constructed according to Wilfling et al.^11^) or PEX30::mRuby, LiveDrop or PEX30 were subcloned into pSBbi-BP vector. Each plasmid was co-transfected into cells that stably expressed SEIP-1::Venus as established above. Selection of stable integrants was facilitated by 10 μg/mL puromycin (InvivoGen) and 0.5 mg/mL hygromycin for at least 7 days. To constitutively express SEIP-1::Venus and inducibly express of SAR1a (H79G)::mRuby, COS7 cells were treated by procedures elaborated as above, but with a mouse *sar1a (H79G)::mRuby* fusion sequence subcloned into the pSBtet-BP backbone.

### Transmission electron microscopy

Chemical staining and dehydration of COS7 cells that stably expressed SEIP-1::V5-APEX2 were performed based on published methods^47,79^. Prior to fixation, cells were incubated with 400 μM oleic acid-BSA complex (Sigma) for 24 h. Fixation was conducted using 2.5% glutaraldehyde (Electron Microscopy Science) in wash buffer (100 mM sodium cacodylate with 2 mM CaCl_2_, pH 7.4). For diaminobenzidine (DAB) staining, 5 mg/mL DAB free base (Sigma) stock solution dissolved in 0.1 M HCl was freshly diluted with 10 mM H_2_O_2_ in cold wash buffer, and cells were incubated in the solution for 10 min. SEIP-1::Venus stable cells were used as the negative control for assessing non-specific DAB deposition. Two percent of osmium tetroxide (Electron Microscopy Science) staining was performed for 40 min in cold wash buffer, followed by incubation with chilled 2% uranyl acetate in ddH_2_O overnight at 4 °C. Infiltration was performed at room temperature with a progressive series (10, 25, 50, 75, and 100%) of EMBED-812 resin to ethanol dilutions. Specimens were incubated for at least 3 h in each dilution. After three changes of 100% Epon resin, samples were then transferred to a plastic mold and cured at 60 °C for 40 h^80^. Cured blocks were then trimmed and sectioned with a diamond knife. In total, 150-nm thin sections were cut and mounted on copper slot grids coated with formvar and further imaged with a Hitachi H-7650 transmission electron microscope operated at 80 kV.

For 3D model reconstruction, serial images were stacked and aligned using IMOD software package (Boulder Laboratory of 3D Electron Microscopy of the Cell, University of Colorado at Boulder). 3D modeling of ER without obvious DAB/APEX2 staining and LDs were performed using 3dmod graphic module while DAB/APEX2 precipitates were reconstructed using the auto-contour function under high contrast with a threshold level.

### Immunostaining endogenous SEIP-1 in dissected *C. elegans*

Animals were dissected, fixed and immunostained according to published methods with minor modifications. Briefly, ~200 L4 larval animals of each genotype were dissected in 1xPBS/0.0005% Triton X-100 (Sigma) with 0.2 mM levamisole for intestine extrusion and fixed for 4 h at 4 °C in 2% paraformaldehyde. At room temperature, the fixed animals were permeabilized for 1 h in 1xPBS/0.1% Triton X-100, followed by a 1 h blocking in 1×PBS/0.1% Tween 20/1% bovine serum albumin (BSA). Primary (1:100 diluted monoclonal anti-FLAG antibody (M2) (Sigma)) and secondary antibody (1:100 diluted Alexa Fluor 488 Donkey anti-Mouse IgG (H + L) (Invitrogen)) incubations were both performed overnight at 4 °C in 1×PBS/0.1% Tween 20/1% BSA. Sample was post-stained by HCS LipidTOX^TM^ red neutral lipid stain (Invitrogen) and Hoechst 33342 (Invitrogen) according to manufacturer’s instructions and mounted onto a 35-mm MatTek glass-bottom dish coated with poly-D-lysine prior to imaging.

### Total lipid extraction

Sample was prepared and extracted according to published methods with slight modifications^41^. For total lipid extraction from bacteria, ~15 mL overnight culture was concentrated 10× and spread on a 10 cm NGM plate. The plate was immediately dried in a laminar flow hood and incubated at 20 °C for 2 days. Bacteria was rinsed off the plate, pelleted and washed three times using 0.9% NaCl solution. Two plates were combined as one sample, at least three samples were analyzed for each bacterial strain. After the last wash, the pellet was resuspended and sonicated in ice water bath for five times at 40% amplitude with a 2 min on/30 s off cycle. One-tenth of the lysates were sampled to determine the soluble protein concentration using the BCA Protein Assay Kit (Pierce) and used for subsequent data normalization. Known amount of palmitic acid-D31 (Sigma) was added to the remaining lysates in order to control for extraction efficiency, sample loss and subsequent trans-methylation efficiency. One volume of the remaining lysates were subsequently extracted using ten volumes of pre-chilled Folch solution (chloroform:methanol, 2:1, v-v) followed by five volumes of chloroform. Total lipids were pooled together from bottom chloroform phases into a clean glass vial with a PTFE cap and dried under a gentle stream of nitrogen at 37 °C.

For FAs analysis of *C. elegans*, ~4000 newly hatched L1 larvae were seeded onto each plate prepared as described above and allowed to grow to the late-L4 stage. L4 larvae were harvested and washed four times with 1×PBS/0.001% Tween 20. Worm pellets were snap- frozen and stored in −80 °C prior to lipid extraction. Each plate of worms was used as one sample, at least four independent samples were used for each condition. For lipid extraction, 500 μl of 1xPBS/0.001% Tween 20/0.001% butylated hydroxytoluene (BHT) was added to each sample pellet and sonicated in ice water bath for 15 times at 40% amplitude with a 2 min on/30 sec off cycle. One hundred microliters of the sonicated sample was mixed with an equal volume of 4× SDS buffer (8% SDS, 200 mM Tris-HCl pH 6.8) and denatured at 95 °C for 10 min. Concentration of soluble protein was determined using the BCA Protein Assay Kit (Pierce) and used for subsequent data normalization. Known amount of heptadecanoic acid (C17:0, Nu-Chek Prep) was added to the remaining lysates in order to control for extraction efficiency, sample loss and subsequent trans-methylation efficiency. The remaining 400 μl worm lysate was subsequently subjected to total lipid extraction as described for bacteria above.

### Preparation of fatty acid methyl esters (FAMEs)

Dried lipids were re-dissolved in 1.95 mL methanol. Trans-methylation was carried out by adding 50 μL sulfuric acid and incubating the sample mixture at 80 °C for 1 h. After adding 1.5 mL Milli-Q water, fatty acid methyl esters (FAMEs) were extracted with 200 or 400 μL n-hexane (RCI Labscan) and transferred into autosampler vials with inserts.

### GC-MS analysis

GC-MS analysis was performed on an Agilent 7890B gas chromatograph equipped with an Agilent 5977 A mass spectrometer, an Agilent 7693 autosampler, and an Agilent DB-23 30 m × 0.25 mm × 0.25 μm GC column (#122-2332; Agilent). One microliter of sample was injected in splitless mode. Carrier gas helium was set at a constant flow rate of 1.6 mL/min. The inlet temperature was 220 °C. The column temperature was maintained at 100 °C for 1 min, and then increased to 180 °C at 10 °C/min, then held at 180 °C for 5 min. Finally, temperature was raised to 220 °C at 5 °C/min, and held at 220 °C for 3 min. The retention time of each FAMEs were identified when compared with the peaks of FAME standards that purchased from Nu-Chek Prep or by using Supelco 37 Component FAME Mix. Mass spectra of unidentified peaks were matched against NIST14 library. The response factors (RFs) for each FAMEs relative to the internal standard were determined from the constructed calibration curve. Quantitation was achieved by relating the peak area of each FAME with the spiked internal standard.

### Reporting summary

Further information on research design is available in the Nature Research Reporting Summary linked to this article.

## Supplementary information

Supplementary Figures

Supplementary Video 1

Supplementary Video 2

Supplementary Video 3

Reporting Summary

## Data Availability

The data that support the findings of this study are available from the corresponding author upon request.
