# Supplementary material for: Dietary fatty acids promote lipid droplet diversity through seipin enrichment in an ER subdomain
Source: Nat Commun. 2019 Jul 1;10:2902. doi: 10.1038/s41467-019-10835-4 (PMC6602954; doi:10.1038/s41467-019-10835-4)

Dietary fatty acids promote lipid droplet diversity through seipin enrichment in an ER subdomain

Cao et al

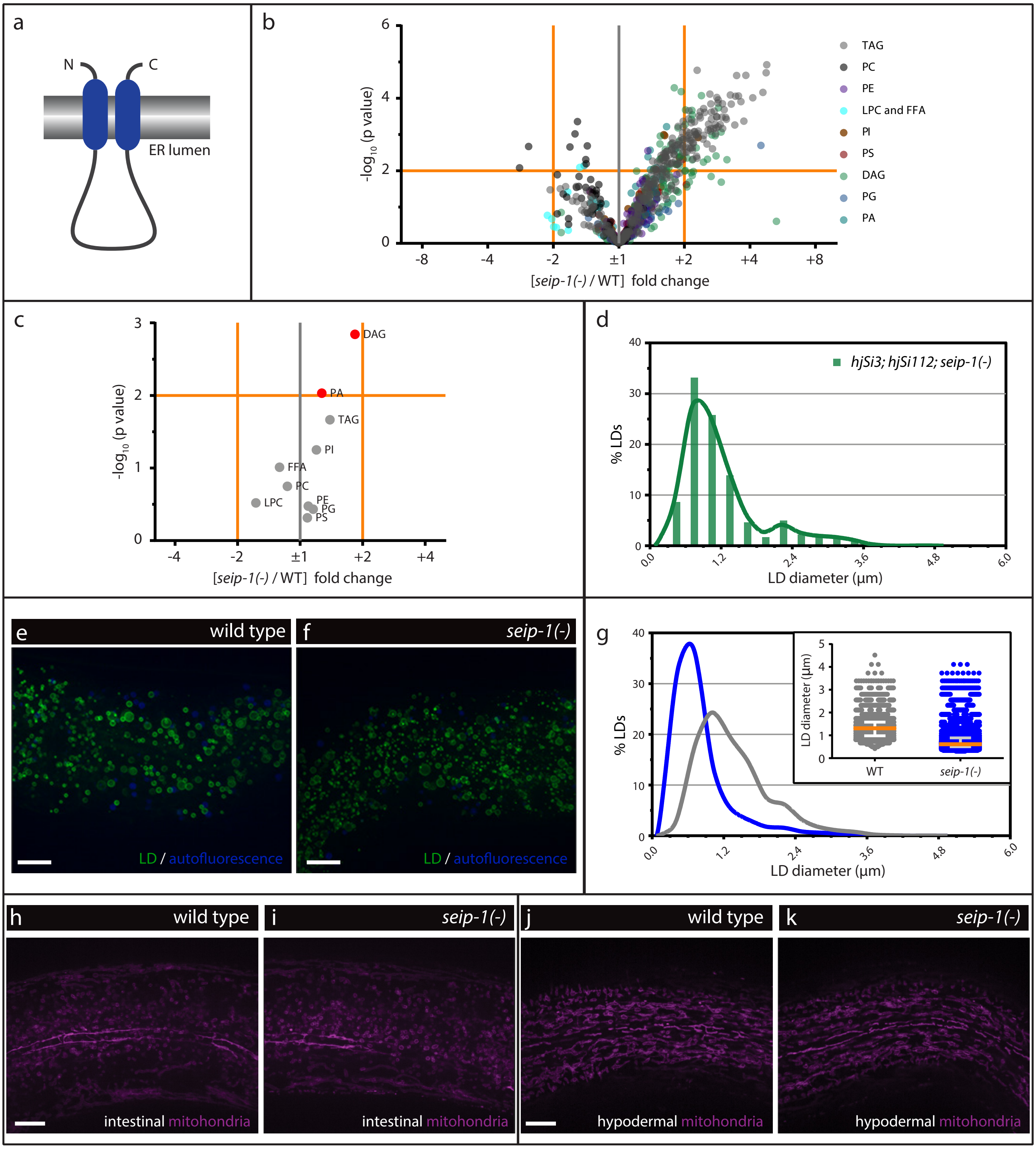

Supplementary Figure 1. Data associated with Fig. 1.
(a) Predicted membrane topology of SEIP-1.
(b) Volcano plot of all 738 lipid species detected in wild-type (WT) or *seip-1(tm4221)* animals.
(c) Volcano plot of total phospholipid (PC, PE, PI, PS, PG, PA and LPC), diacylglycerol (DAG), triacylglycerol (TAG) and free fatty acid (FFA) in WT or *seip-1(tm4221)* animals.
(d) An example of the curve fitting model used in **Fig. 1e**, **2h**, **5c** and **6g**. The curve was fitted twice using Fit Spline/LOWESS (20 points in smoothing window, 4000 segments) method in GraphPad Prism based on a histogram with a bin size of 0.3µm.
(e) Visualization of LDs using the marker DHS-3::GFP (*hj120*, a knock-in allele at the endogenous *dhs-3* locus) in a WT larval L4 stage animal. Autofluorescence from lysosome related organelles (LROs) is pseudocolored blue. A projection of 4.5µm z stack centering at the second intestinal segment is shown.
(f) As in **(e)**, but with a *seip-1(tm4221)* mutant animal.
(g) Frequency distribution of LD diameter. The curve was fitted using the same method as in **(d)**. Inset: a scatter plot showing the median and inter-quartile range of LD size. Total number of LDs measured: WT = 2,423; *seip-1(tm4221)* = 8,119.
(h) Visualization of intestinal mitochondria using the marker TOMM-20 (N- terminus)::mRuby (*hjSi489*) in a WT larval L4 stage animal. mRuby signals were pseudocolored magenta. A projection of 3µm z stack centering at the second intestinal segment is shown.
(i) As in **(h)**, but with a *seip-1(tm4221)* mutant animal.
(j) As in **(h)**, but with a single focal plane showing the morphology of hypodermal mitochondria.
(k) As in **(j)**, but with a *seip-1(tm4221)* mutant animal.
For all fluorescencen images, scale bar = 10µm.

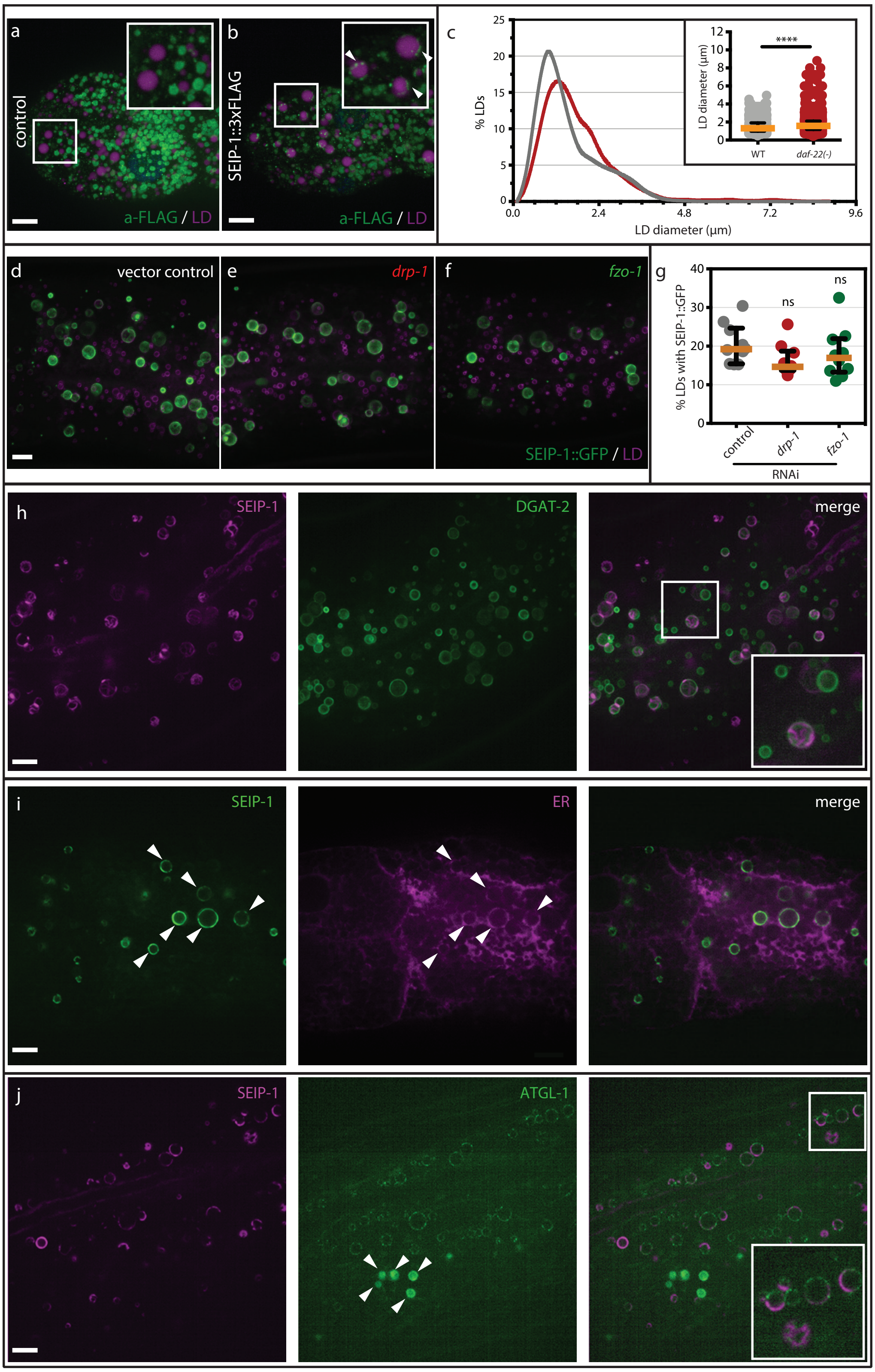

Supplementary Figure 2. Data associated with Fig. 2.
(a) A dissected wild-type (WT) control animal was fixed and immunostained with mouse anti-FLAG antibodies and Alexa Fluor 488 anti-Mouse IgG. LDs were stained with LipidTox Red and pseudocolored magenta. Non-specific signals from the secondary antibodies are shown in green. Scale bar = 10µm. The boxed region was magnified 2x and shown in the inset.
(b) As in **(a)**, but with a *seip-1::3xFLAG* knock-in allele. Specific signals in the proximity of LDs are indicated by white arrowheads.
(c) Frequency distribution comparison of LD diameter between WT and *daf-22(ok693)* animals. The curve was fitted as in **Supplementary Figure 1d**. Inset: a scatter plot of LD diameter. Total number of LDs measured: WT = 1,115; *daf- 22(ok693)* = 1,216.
(d) Visualization of SEIP-1::GFP (*hjSi3*) in a larval L4 stage WT animal with control RNAi knockdown. A LD marker mRuby::DGAT-2 (*hjSi112*) was used and the mRuby signal is pseudocolored magenta. Projections of 4.5µm z stack centering at the second intestinal segment are shown. Scale bar = 5µm.
(e) As in **(d)**, but with ubiquitous RNAi knockdown of *drp-1*, which encodes a dynamin-related protein required for mitochondrial fission.
(f) As in **(d)**, but with ubiquitous RNAi knockdown of *fzo-1*, which encodes a mitofusin ortholog required for mitochondrial fusion.
(g) A scatter plot summarizing the percentage of LDs associated with SEIP-1::GFP in the second intestinal segment in animals with control knockdown or ubiquitous knockdown of *drp-1* or *fzo-1*. n = 10 individual animals.
(h) Visualization of SEIP-1::tagRFP (*hjSi434*) in a larval L4 stage animal. A projection of 3µm z stack centering at the second intestinal segment is shown. The LD marker GFP::DGAT-2 (*hjSi56*) is used. The tagRFP signal is pseudocolored magenta. The boxed region was magnified 2x and shown in the inset. Scale bar = 5µm.
(i) Visualization of SEIP-1::GFP (*hjSi3*) in a larval L4 stage animal. A single focal plane centering at the second intestinal segment is shown. A luminal ER mCherry marker (*hjSi158*) is used and pseudocolored magenta. Arrows point to the cages adjacent to the luminal ER signals. Scale bar = 5µm.
(j) Visualization of SEIP-1::tagRFP (*hjSi434*) and ATGL-1::GFP in a larval L4 stage animal. A single focal plane centering at the second intestinal segment is shown. The tagRFP signal is pseudocolored magenta. The GFP fluorescence is weaker than autofluorescence from lysosome related organelles (indicated by arrowheads). The boxed area was magnified 2x and shown in the inset. Scale bar = 5µm.
For all scatter plots, median with interquartile range is displayed.

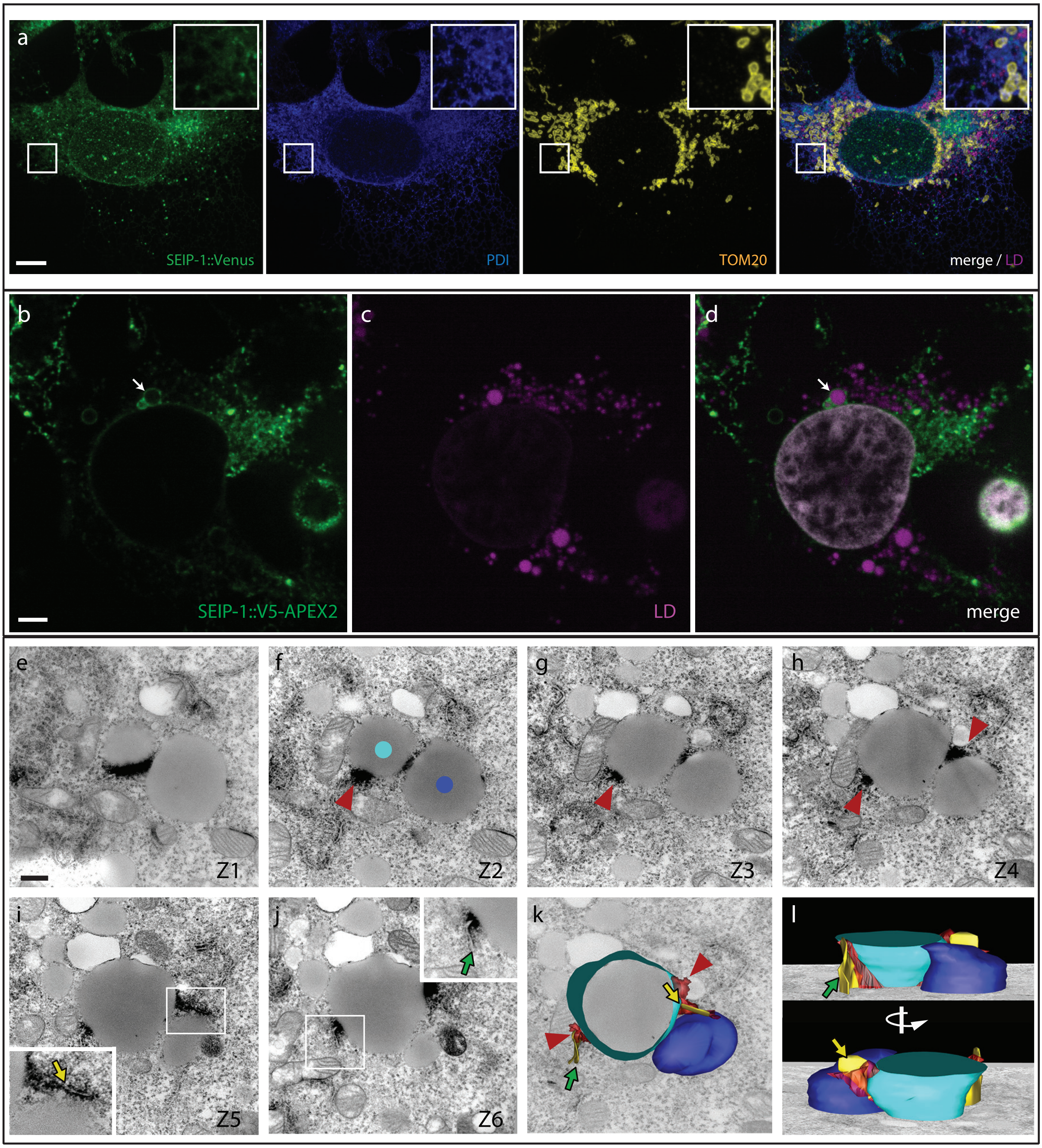

Supplementary Figure 3. Data associated with Fig. 3.
(a) The SEIP-1::Venus protein was stably expressed in COS7 cells. The cells were incubated with EtOH (vehicle control) instead of oleic acid (OA) in **Fig. 3a**, fixed and immunostained for endogenous PDI (the ER) and TOM20 (mitochondria). Alexa Fluor (AF)-405 and 594 conjugated secondary antibodies were used for visualising PDI and TOM20, respectively. AF594 signals were pseudocolored yellow. FAS was used to stain LDs and pseudocolored magenta. A projection of 4.5μm Z stack is shown. The boxed area was magnified 3x and shown in the inset. Scale bar = 10μm.
(b) COS7 cells stably expressing SEIP-1::V5-APEX2 as in **Fig. 3e-g** were subjected to immunostaining with anti-V5 antibodies. The cells were incubated with OA prior to fixation. The white arrow indicates an enlarged LD surrounded by SEIP-1. Scale bar = 5μm.
(c) As in **(b)**, but with LDs stained with the lipid dye FAS (pseudocolored magenta).
(d) As in **(b)**, but with SEIP-1, LD and Hoechst 33342 (pseudocolored grey) signals merged.
(e-l) Transmission electron micrographs of six consecutive sections (150nm thick) of a COS7 cell stably expressing a SEIP-1::V5-APEX2 fusion protein. Dark deposits that decorate SEIP-1 positive ER membranes immediately adjacent to LDs are marked in **(f)**, **(g)**, and **(h)** with red arrowheads. The insets in **(i)** and **(j)** show 2x magnification of the boxed areas in which dark deposits decorate SEIP-1 positive ER membrane extensions distal to LDs (arrows). The micrographs were captured at 25,000x and the scale bar in **(e)** indicates 0.5μm. A three-dimensional model in **(l)** was generated from the serial sections shown in **(e-j)**. Two LDs (cyan and blue dots in **(f)**), ER membrane extensions (yellow and green arrows in the insets of **(i)** and **(j)**, respectively) and ER membranes proximal to LDs (red arrowheads) are shown. **(l)** Side views of the model in **(k)** is shown. The bottom panel shows the model after 180° rotation along the vertical axis. The ER does not appear as tubules in the reconstruction because of the thickness of the sections.

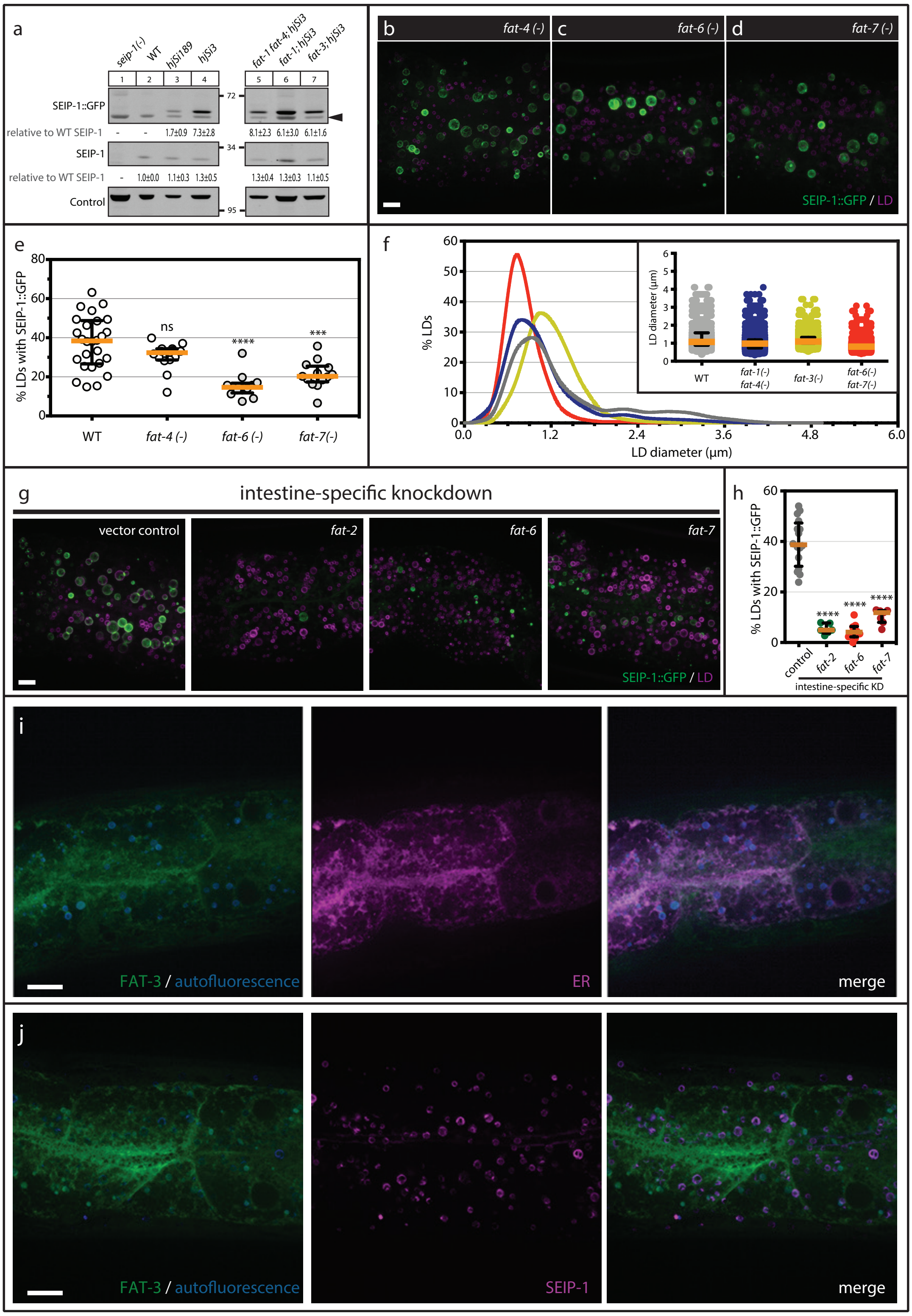

Supplementary Figure 4. Data associated with Fig. 4.

(a) The expression levels of SEIP-1::GFP and endogenous SEIP-1 were determined by SDS- PAGE and immunoblotting with anti-SEIP-1 antibodies. A non-specific band at ~95kDa served as a loading control. The expression level of endogenous SEIP-1 in wild-type (WT) animals served as a reference for normalization of signals in other samples. The mean ± SD from three independent experiments (including the blot in **Fig. 1d**) is shown. The arrowhead indicates a non-specific band.
(b) Visualization of SEIP-1::GFP (*hjSi3*) in a *fat-4 (wa14)* mutant larval L4 stage animal. A projection of 4.5μm z stack centering at the second intestinal segment is shown. The LD marker mRuby::DGAT-2 (*hjSi112*) was used and the mRuby signal is pseudocolored magenta. Scale bar = 5μm.
(c) As in **(b)**, but with a *fat-6 (tm331)* mutant animal.
(d) As in **(b)**, but with a *fat-7 (wa36)* mutant animal.
(e) The percentage of LDs associated with SEIP-1::GFP. No. of animals analysed: WT = 26, *fat-4(-)* = 14, *fat-6(-)* = 12, *fat-7(-)* = 13.
(f) Frequency distribution comparison of LD diameter between WT, *fat-1(wa9) fat-4(wa14)*, *fat-3(ok1126)* and *fat-6 (tm331);fat-7 (wa36)* animals. The curve was fitted as in **Supplementary Figure 1d**. The fluorescence images were displayed as in **Fig. 4b, e** and **f**. Inset: a scatter plot summarizing LD diameter. Total number of LDs measured: WT = 1,520, *fat-1(wa9) fat- 4(wa14)* = 2,786, *fat-3(ok1126)* = 5,370, *fat-6 (tm331);fat-7 (wa36)* = 3,594.
(g) Visualization of SEIP-1::GFP (*hjSi3*) with control RNAi knockdown or intestine-specific knockdown of *fat-2*, *fat-6* or *fat-7* in larval L4 stage animals. Since *fat-6* and *fat-7* cDNAs share high sequence-similarity, specific knockdown of either *fat-6* or *fat-7* cannot be achieved (gene expression after knockdown summarized in **Supplementary Figure 6k**. A LD marker mRuby::DGAT-2 (*hjSi112*) was used and the mRuby signal is pseudocolored magenta. Projections of 4.5μm z stack centering at the second intestinal segment are shown. Scale bar = 5μm.
(h) The percentage of LDs associated with SEIP-1::GFP when *fat-2*, *fat-6* or *fat-7* was knocked down specifically in the intestine as in **(g)**. No. of animals analysed: control = 16, *fat-2* = 6, *fat-6* = 10, *fat-7* = 7.
(i) Visualization of FAT-3::GFP (*hjSi222*) in a larval L4 stage *fat-3(ok1126)* animal. The *hjSi222* transgene rescues the *fat-3* mutant phenotypes. A projection of 3μm z stack centering at the second intestinal segment is shown. Autofluorescence from lysosome related organelles is pseudocolored blue. The ER is marked by a tail-anchored mRuby red fluorescent protein that is expressed exclusively in the intestine (*hjSi127*). The mRuby signal is pseudocolored magenta. Scale bar = 10μm.
(j) Visualization of both FAT-3::GFP (*hjSi222*) and SEIP-1::tagRFP (*hjSi434*) in a WT larval L4 stage animal. A projection of 2μm z stack centering at the second intestinal segment is shown. Autofluorescence from lysosome related organelles is pseudocolored blue. The tagRFP signal is pseudocolored magenta. Scale bar = 10μm.
For all statistics, ***p < 0.001; ****p < 0.0001 (unpaired t-test). For all scatter plots, median with interquartile range is displayed.

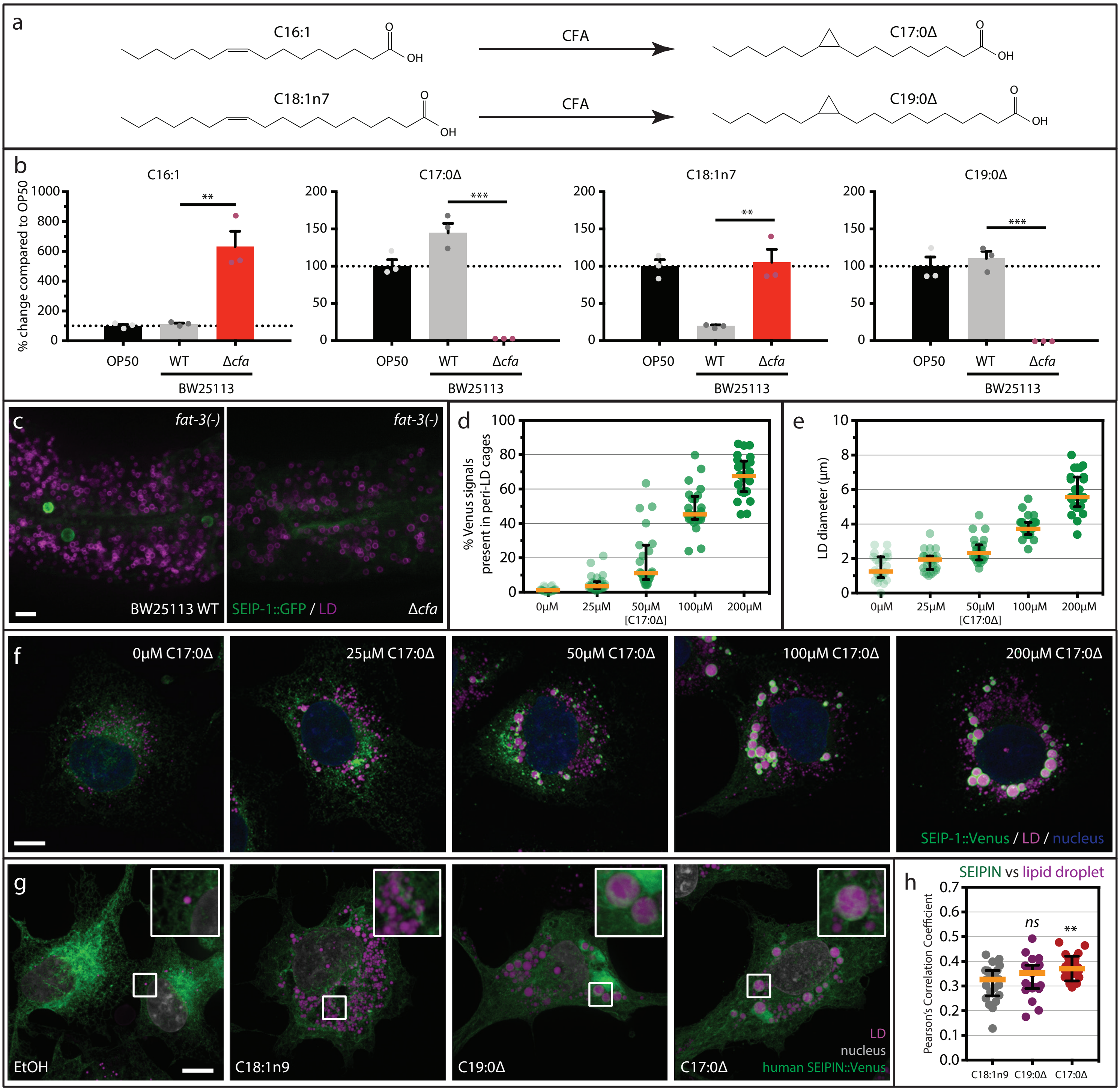

Supplementary Figure 5. Data associated with Fig. 5.

(a) The biosynthetic pathway for cyclopropane fatty acids in *Escherichia coli*.

(b) The normalized abundance of cyclopropane fatty acids and their mono-unsaturated precursors in OP50, BW25113 WT and BW25113 Δ*cfa E. coli*. Mean ± SEM from 3 independent samples is shown.

(c) Visualization of SEIP-1::GFP (*hjSi3*) in a *fat-3 (ok1126)* mutant larval L4 stage animal fed either WT or Δ*cfa E. coli* BW25113. mRuby::DGAT-2 (*hjSi112*) was used as the LD marker and mRuby signals are pseudocolored magenta. A projection of 4.5μm z stack centering at the second intestinal segment is shown. Scale bar = 5μm.

(d) The effects of cis-9,10-methylenehexadecanoic acid (C17:Δ) supplementation on the percentage of Venus signals at peri-LD cages in COS7 cells stably expressing SEIP- 1::Venus. The fluorescence images are displayed in **(f)**. No. of cells analysed: 0mM = 24, 25mM = 24, 50mM = 23, 100mM = 24, 200mM = 25 (also applies to **(e)**).

(e) As in **(d)**, but the effect of C17:Δ supplementation on the maximum LD diameter is shown.

(f) COS7 cells stably expressing SEIP-1::Venus were supplemented with increasing concentration of C17:0Δs. LDs were stained with LipidTox Red (pseudo-colored magenta) and nuclei were stained by Hoechst 33342 (in blue). A projection of 4.5μm z stack is shown. Scale bar = 10μm.

(g) COS7 cells stably expressing human seipin isoform 2::Venus were supplemented with either EtOH vehicle, oleic acid (OA, C18:1n9), 11,12-methylene octadecanoic acid (C19:0Δ) or 9,10-methylene hexadecanoic acid (C17:0Δ). Cells were incubated with 800μM FAs for 6h. GLA (C18:3n6) was not tested due to its toxicity at high concentrations. LDs were stained with LipidTox Red (pseudocolored magenta) and nuclei were stained by Hoechst 33342 (pseudocolored grey). A projection of 4.5μm z stack is shown. Boxed regions were magnified 3x and shown in the inset. Scale bar = 10μm.

(h) Colocalization coefficient between human seipin::Venus and lipid droplets signals calculated from 4.5μm z-stacked volumes. No. of cells analysed: C18:1n9 = 22, C19:0Δ = 21, C17:0Δ = 23.

For all statistics, **p < 0.01; ***p < 0.001 (unpaired t-test). For all scatter plots, median with interquartile range is displayed.

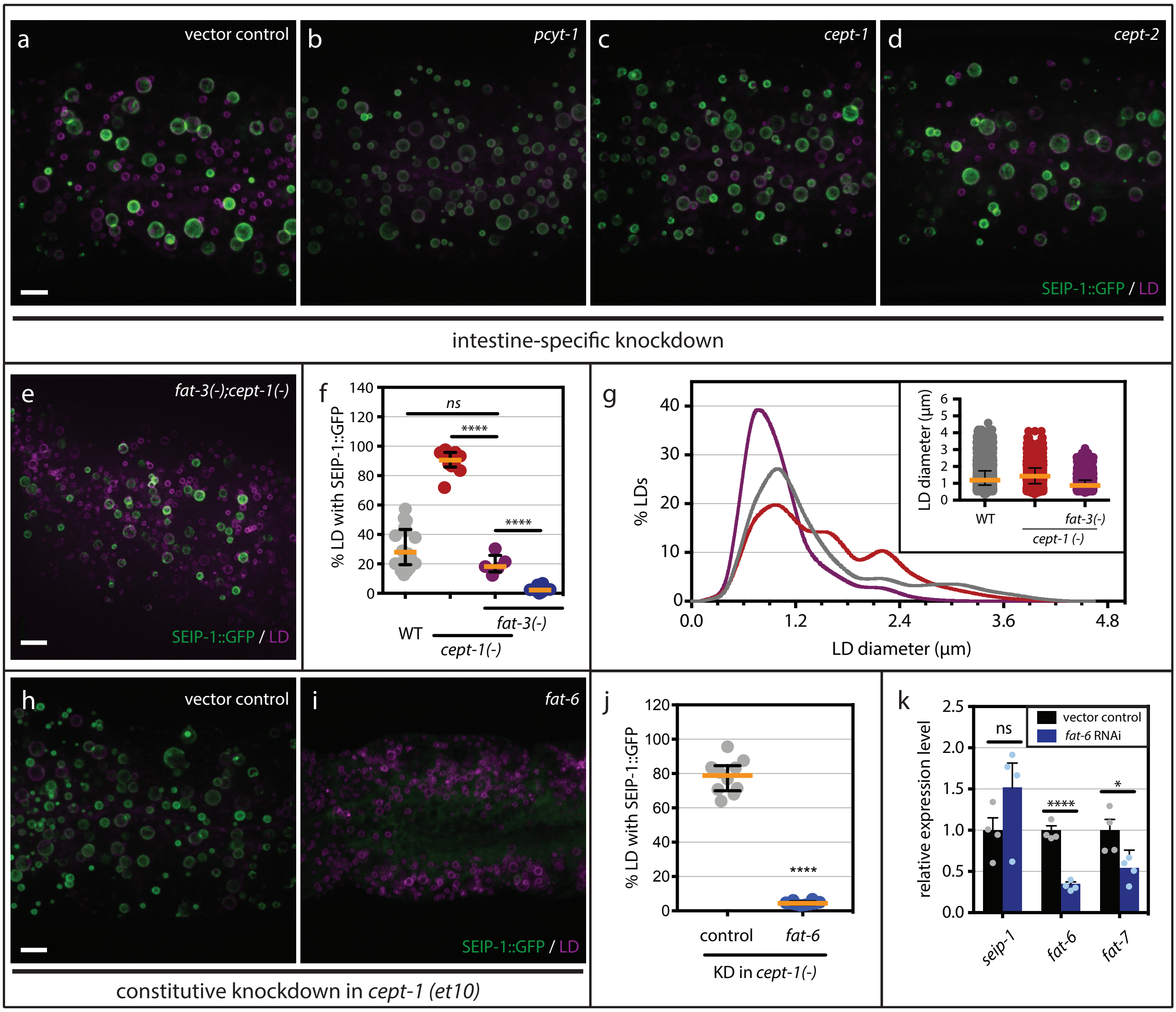

Supplementary Figure 6. Data associated with Fig. 6.

(a) Visualization of SEIP-1::GFP (*hjSi3*) in a larval L4 stage wild-type (WT) animal with control knockdown. A LD marker mRuby::DGAT-2 (*hjSi112*) was used and the mRuby signal is pseudocolored magenta. Projections of 4.5μm z stack centering at the second intestinal segment are shown. Scale bar = 5μm.

(b) As in **(a)**, but with intestine-specific RNAi knockdown of *pcyt-1*, which encodes a phosphocholine cytidylyltransferase catalysing the second last step of *de novo* phosphatidylcholine (PC) synthesis.

(c) As in **(a)**, but with intestine-specific RNAi knockdown of *cept-1*, which encodes a choline/ethanolamine phosphotransferase catalysing the last step of *de novo* PC synthesis.

(d) As in **(a)**, but with intestine-specific RNAi knockdown of *cept-2*, which is paralogous to *cept-1*.

(e) As in **(a)**, but with a larval L4 stage *fat-3(ok1126);cept-1(et10)* mutant animal.

(f) The percentage of LDs associated with SEIP-1::GFP in indicated mutant backgrounds. No. of animals analysed: WT = 18, *cept-1(-)* = 10, *cept-1(-);fat-3(-)* = 5, *fat-3(-)* = 12.

(g) Frequency distribution comparison of LD diameter between WT, *cept-1(et10)* and *fat- 3(ok1126);cept-1(et10)* animals. The curve was fitted as in **Supplementary Figure 1d**. Inset: a scatter plot summarizing LD diameter. Total number of LDs measured: WT = 2,347, *cept-1 (et10)* = 1,306, *fat-3(ok1126);cept-1(et10)* = 1,360.

(h) As in **(a)**, but with a larval L4 stage *cept-1(et10)* animal with control knockdown.

(i) As in **(h)**, but with ubiquitous RNAi knockdown of *fat-6*.

(j) The percentage of LDs associated with SEIP-1::GFP. n = 10 individual animals.

(k) Reduction of *fat-6* and *fat-7* gene expression levels in larval L4 WT animals that had been treated with *fat-6* RNAi. The gene expression levels were measured by real-time PCR and normalized against the mean expression level of the vector control group. Mean ± SEM from 4 independent samples is shown for each gene.

For all statistics, *$p < 0.05$; ****$p < 0.0001$ (unpaired t-test). For all scatter plots, median with interquartile range is displayed.

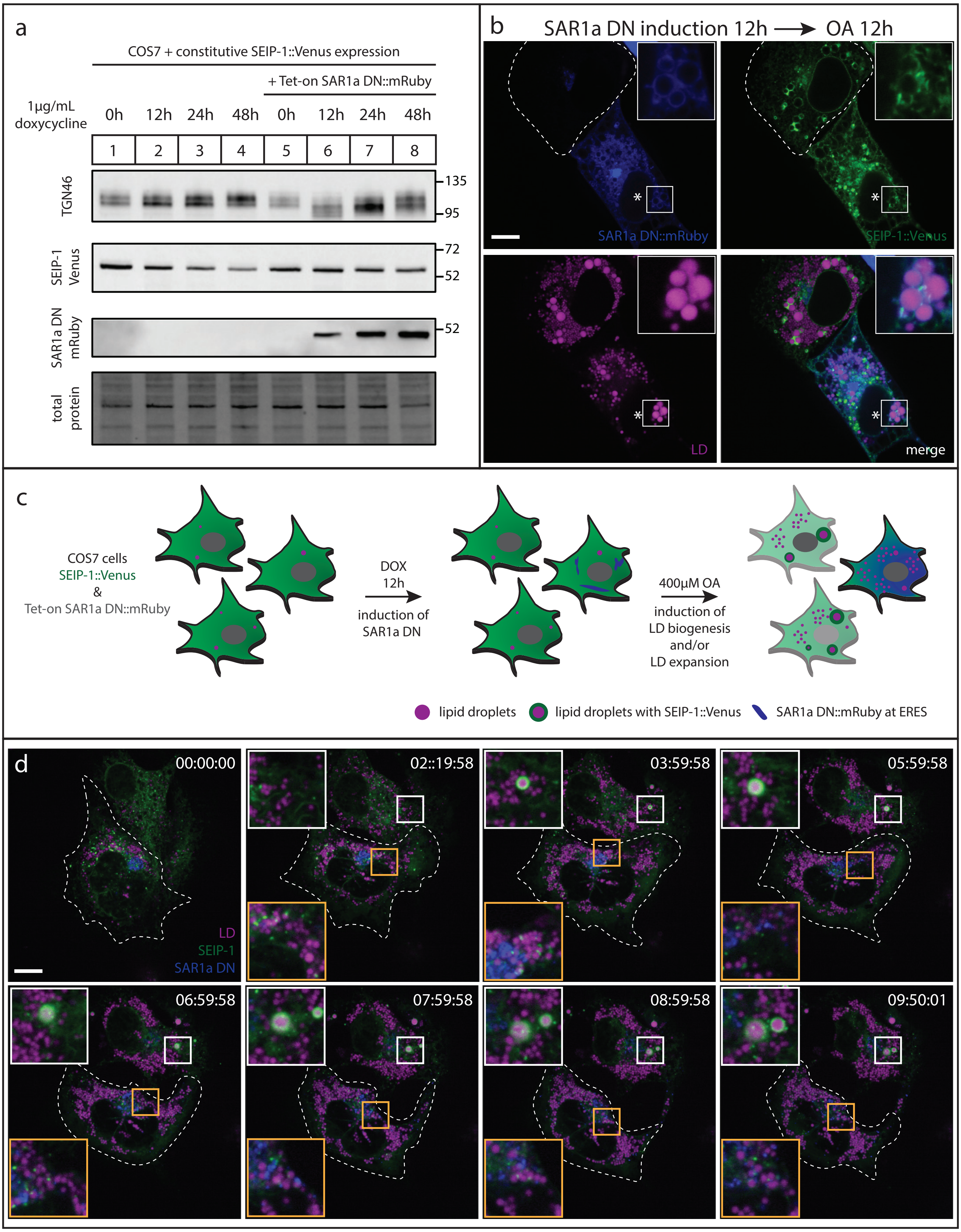

Supplementary Figure 7. Data associated with Fig. 7.

(a) The expression levels of TGN46, SEIP-1::Venus, SAR1a H79G (DN)::mRuby were determined by SDS- PAGE and immunoblotting over the time course of doxycycline treatment. Total protein levels visualized in a TGX stain-free gel (Bio-rad) is used as the loading control and shown in the bottom panel. The down-shifted TGN46 (lanes 6 and 7) represented its less-glycosylated forms, as a result of SAR1a(DN)::mRuby induction.

(b) Overexpression of SAR1a(DN)::mRuby in cells used in **Fig. 7a** and lanes 5-8 of **Supplementary Figure 7a**. The cells were pre-treated with 12-hour of 1µg/mL doxycycline to inhibit COPII budding. They were subsequently loaded with 400µM oleic acid (OA) for another 12 hours before fixation. Lipid droplets were stained by FAS and pseudocolored magenta, mRuby signals are pseudocolored blue (same for all other image panels). Boxed regions were magnified 3x and shown in the insets. Single focal planes are shown. Asterisks mark the cells that overexpressed the SAR1a(DN) mutant, while the adjacent cells with relatively-low level of SAR1a(DN) expression are outlined by dashed lines. Scale bar = 10µm (same for all other image panels).

(c) The experimental scheme used in **(b)** and **(d)**.

(d) The 10-hour live-cell imaging started upon the addition of 400µM OA. Images were captured at 10-minute intervals. Dotted lines mark the cell with detectable SAR1a(DN)::mRuby expression at T0, in contrast to the adjacent cell. Boxed regions were magnified 3x and shown in the insets.

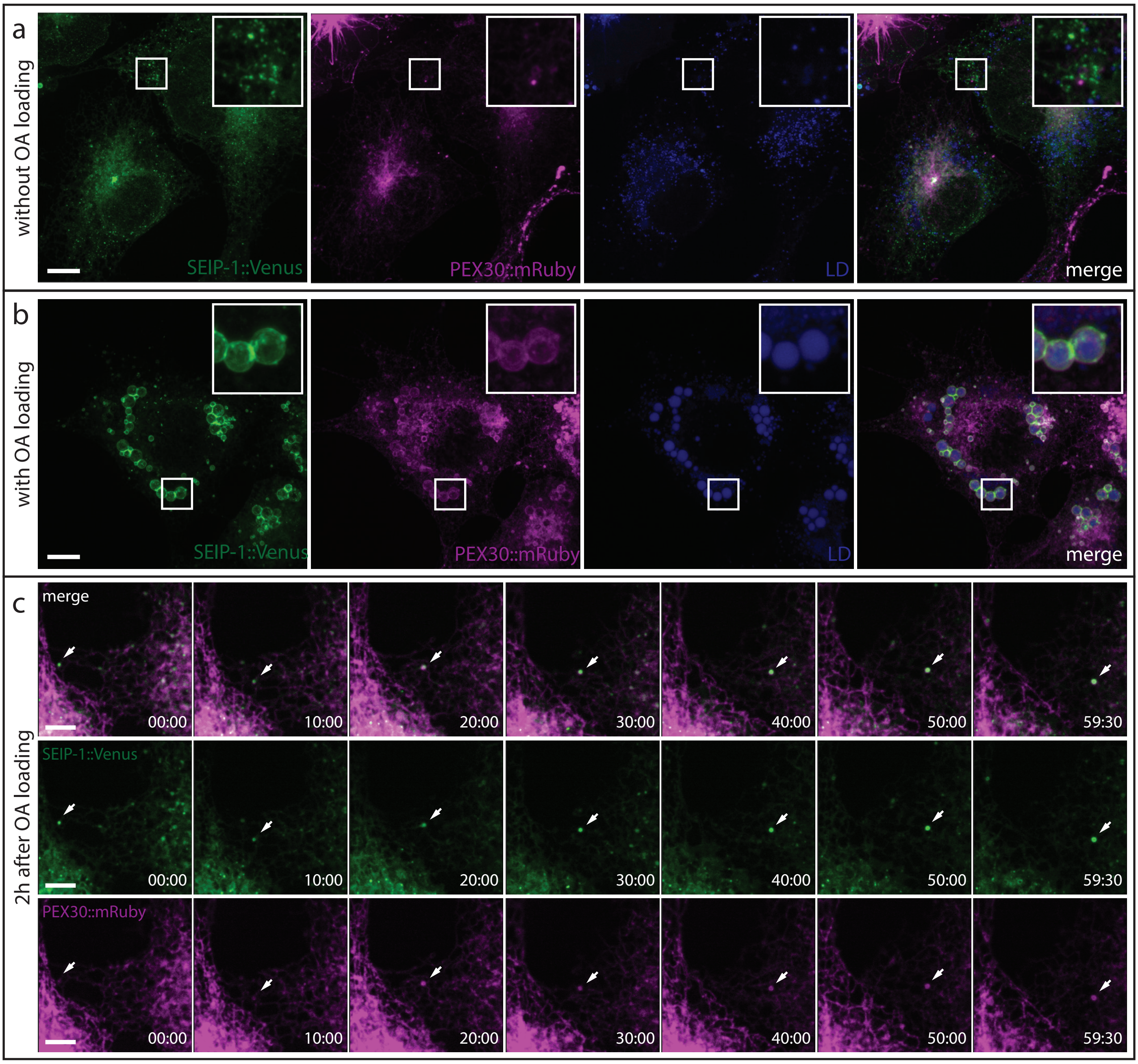

Supplementary Figure 8. SEIP-1 and PEX30 mark the same ER domain upon oleate loading.
(a) The SEIP-1::Venus protein and a budding yeast peroxisome biogenesis marker, PEX30::mRuby were stably co-expressed in COS7 cells. The cells were treated with EtOH (vehicle) for 12 hours as a control to **(b)**. Lipid droplets were stained by FAS and pseudocolored blue, mRuby signals are pseudocolored magenta. Boxed regions were magnified 3x and shown in the insets. Scale bar = 10μm.
(b) As in **(a)**, but with cells loaded with 400μM OA for 12 hours before fixation.
(c) Live-cell tracking of SEIP-1::Venus and PEX30::mRuby after OA loading. The cells were pre-incubated with oleic acid for 1.5 hour before live imaging for another 1 hour at 30- second intervals. Arrows marked the same SEIP-1 (+) cage and showed enrichment of both SEIP-1::Venus and PEX30::mRuby signals over time. Scale bar = 3μm.

Supplementary Figure 9. An uncropped version of the blot presented in Fig. 1d.

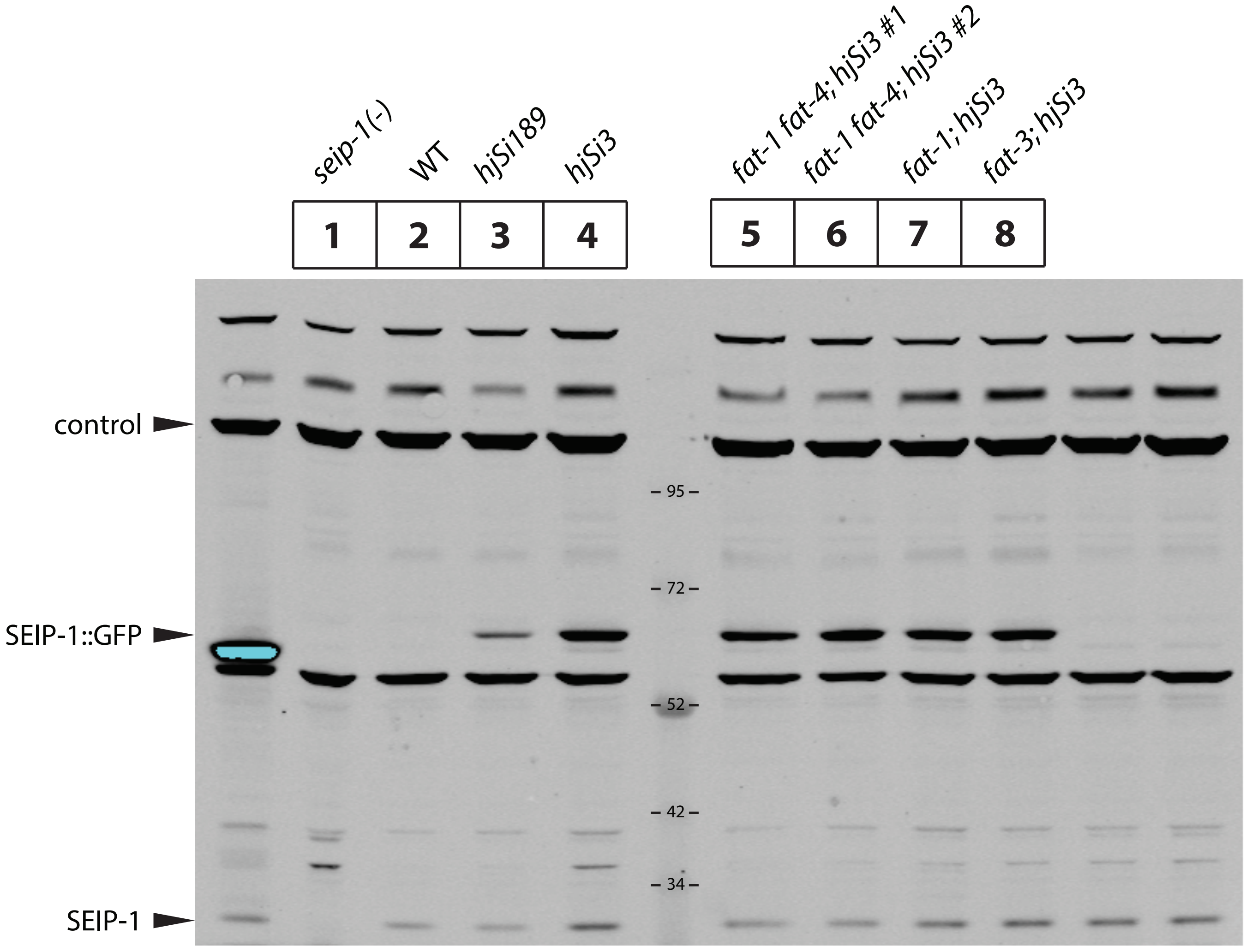

Supplement: Supplementary file 1 — Supplementary Figures [file 41467_2019_10835_MOESM1_ESM.pdf]
